# Current Research Status, Key Technologies, and Future Prospects of High-Flame-Retardant, Low-Powdering Phenolic Boards

**DOI:** 10.3390/polym18161948

**Published:** 2026-08-09

**Authors:** Yifan Zhong, Xinfang Wang, Jinglong Fang, Yonghao Liu, Mingli Liu, Hui Li, Yekai Song, Yong Wang, Jinguo Li

**Affiliations:** 1 College of Chemistry and Chemical Engineering, Dezhou University, Dezhou 253023, China; 2 Shandong Provincial Key Laboratory of Monocrystalline Silicon Semiconductor Materials and Technology, Dezhou 253023, China; 3 Shandong Zhongwei Air Conditioning Equipment Group Co., Ltd., Dezhou 253023, China

**Keywords:** phenolic resin, flame retardancy, resistance to chalking, char structure, thermal safety

## Abstract

Phenolic panels are widely used in applications requiring stringent fire resistance due to their advantages of flame retardancy, heat resistance, low smoke emission, and low toxicity. However, traditional products are prone to powdering and slagging under high temperatures or open flames, leading to structural failure and limiting their use in high-end applications. Currently, there is a lack of comprehensive research summaries and recommendations addressing the critical failure mode known as “carbonization.” This is a review of research progress in this field: first, it analyzes the thermal degradation mechanisms underlying powdering; then, it summarizes key strategies for enhancing anti-powdering performance from two broad categories: chemical strategies (resin modification and flame retardant systems) and physical strategies (interface design and process optimization); next, it outlines relevant characterization methods; finally, it discusses future development trends, aiming to provide a concise reference for related research and development efforts.

## 1. Introduction

### 1.1. The Development History and Current Applications of Phenolic Materials

**Figure 1:**
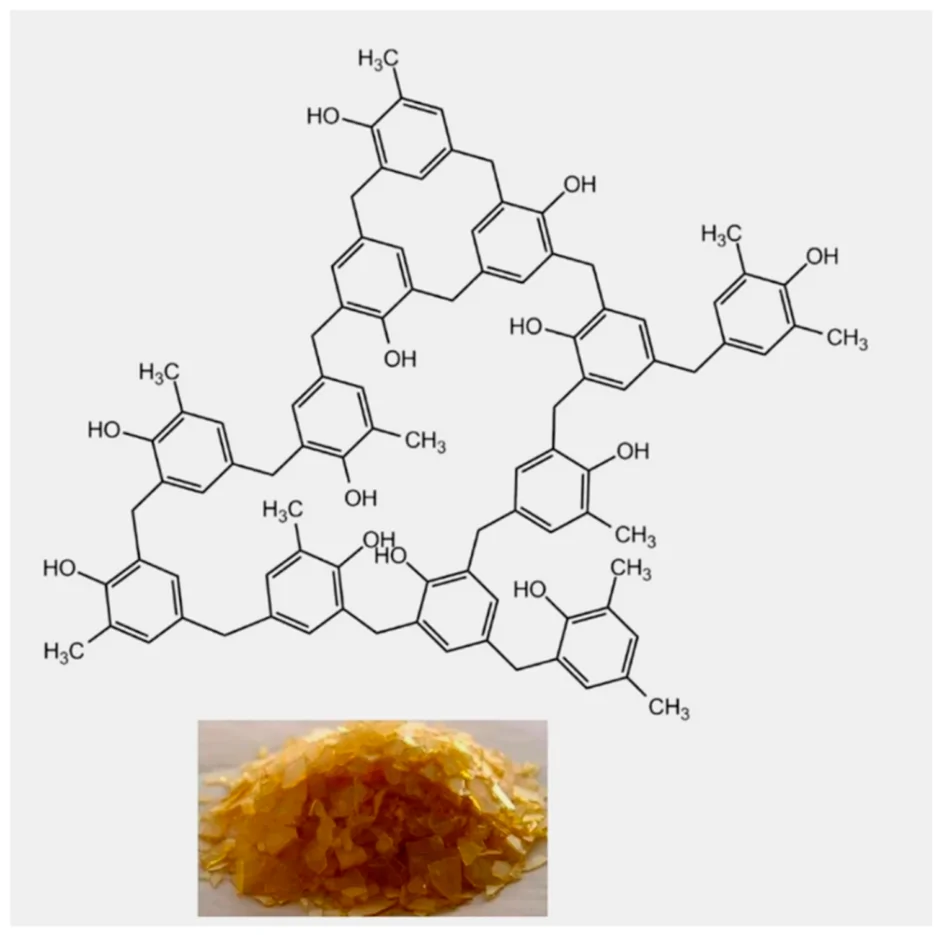
Cross-linking reaction model and photograph of phenolic resin [2].

Phenolic resin (PF), first synthesized in 1907 [1], forms a three-dimensional cross-linked network upon curing, providing exceptional heat resistance, dimensional stability, and inherent flame retardancy (Figure 1). As a matrix combined with fillers, reinforcing fibers, and blowing agents, PF has become an indispensable fire-resistant material in construction, transportation, power electronics, and military applications.

In construction, phenolic boards serve as exterior wall insulation and fire barriers (typically meeting [3]). In transportation, they are used for interior panels and bulkheads in trains, ships, and aircraft. In the power industry, PF panels serve as cable trays and as components of transformer insulation [4]. Additionally, PF composites address fire safety needs in military applications, including ammunition packaging and propellant storage [5], where their low-smoke, low-powdering characteristics are essential. More recently, the possibility of a new flame-retardant mode of action beyond the classical halogen and phosphorus systems has also been shown [6]. Increasingly stringent fire safety regulations worldwide (e.g., [3,7,8]) and the “carbon peak and carbon neutrality” strategy demand that fire-resistant materials not only suppress fire spread but also maintain structural integrity under prolonged thermal exposure—preventing failure due to chalking, delamination, or disintegration.

### 1.2. Technical Challenges in Traditional Phenolic Board Production: Chalking and Its Hazards

Although phenolic resin itself has a very high limiting oxygen index (LOI, usually over 35%) and is classified as a flame-retardant material, once the temperature exceeds its thermal decomposition threshold (generally between 400 °C and 600 °C), the cured network structure will undergo complex thermal oxidative degradation reactions.

During this process, low-molecular-weight gases such as phenol, formaldehyde, water, carbon monoxide, and carbon dioxide are released rapidly. Simultaneously, the carbon skeleton is depleted through oxidation. Ultimately, the process results in the formation of a loose, porous char layer on the surface of the material, which is mechanically weak.

Charring is when the leftover char layer on phenolic foam boards cracks, peels off, or even turns into powder. This happens because of heat stress or outside forces when they are hit by high-temperature flames or thermal radiation. This is a major roadblock that keeps these boards from being used in top-tier fire protection fields. The dangers are pretty serious: first, the fire protection and insulation fail. Once that char layer peels away, the inner layers are directly exposed to flames, which causes the fire resistance to drop quickly. Second, there is a risk of secondary disasters. Those high-temperature carbon particles can become flying embers, the carbon dust pollutes the environment, and it can damage equipment and hurt people’s lungs. Third, it creates structural safety issues. Losing load-bearing parts might lead to instability or even collapse. And fourth, it seriously limits where these materials can be used in high-end applications, like nuclear power plants, deep-sea drilling platforms, or spacecraft. Because of this, it is difficult to meet the needs for long-term fire resistance and harsh conditions, which holds back phenolic foam materials from moving into more advanced applications.

### 1.3. Literature Gap and Contributions of This Review

There are quite a few review articles that have talked about the flame retardancy of phenolic resins. But none of them specifically focus on the phenomenon of “powdering". This powdering issue is actually a critical problem that prevents highly flame-retardant phenolic boards from being used in practical applications. Existing review articles generally discuss the overall thermal degradation of materials, how flame retardants work, or provide a general introduction to applications. However, they do not systematically link the molecular-level char layer structure to the macroscopic powdering behavior. Moreover, these articles do not provide a comprehensive and critical evaluation of anti-powdering strategies from different perspectives, such as chemical modification, flame retardant mode of action, interface engineering, and process control. This review aims to fill this gap. It will be the first to provide a comprehensive, mechanism-based analysis of the powdering issue in PF-based composites.

The main contributions of this work are as follows: (1) establishing a clear causal chain from char microstructure (amorphous carbon, high *I_D_*/*I_G_* ratio) to macro-scale chalking failure; (2) critically comparing chemical and physical anti-chalking strategies, identifying which approaches offer the most promise and which face the greatest challenges; (3) evaluating the adequacy of current characterization and testing methods for anti-chalking performance and highlighting the urgent need for standardization; and (4) proposing a roadmap for future research that integrates computational design, multi-scale evaluation, and green manufacturing principles.

## 2. Analysis of the Flame-Retardant Mode of Action and the Nature of Chalking in Phenolic Resins

### 2.1. Intrinsic Flame-Retardant Properties of Phenolic Resins

The flame-retardant properties of phenolic resins are primarily attributed to their unique chemical structure and thermal behavior, which fall under the condensation-phase flame-retardant mode of action [9,10,11,12,13]. Because phenolic resins have a high amount of aromatic rings in their structure, their molecular backbone is packed with benzene ring structures. The C–C and C–H bonds have very strong bond energies, so they need significantly more energy to break during burning. After curing, the three-dimensional cross-linked network that forms strongly limits how the polymer chains can move, and it reduces the production of volatile flammable products. When phenolic resins are heated, they go through aromatization, condensation, and cyclization reactions. This leads to a leftover carbon content that can reach 55% to 70% in a nitrogen atmosphere at 800 °C. This surface carbon layer does three important things: first, it works as a thermal barrier by blocking heat from penetrating into the material underneath; second, it acts as an oxygen barrier, since its dense structure limits oxygen coming in and volatile gases going out; and third, it serves as a mass transfer barrier, slowing down the release of liquid breakdown products from the polymer.

### 2.2. An In-Depth Analysis of the Underlying Processes of Powdering

The term “powdering” refers to the mechanism by which the carbon layer on the material’s surface fails under the combined effects of thermochemical and other physical forces. The fundamental reason is that the carbon layer formed by traditional phenolic resins has structural defects in itself.

**Figure 2:**
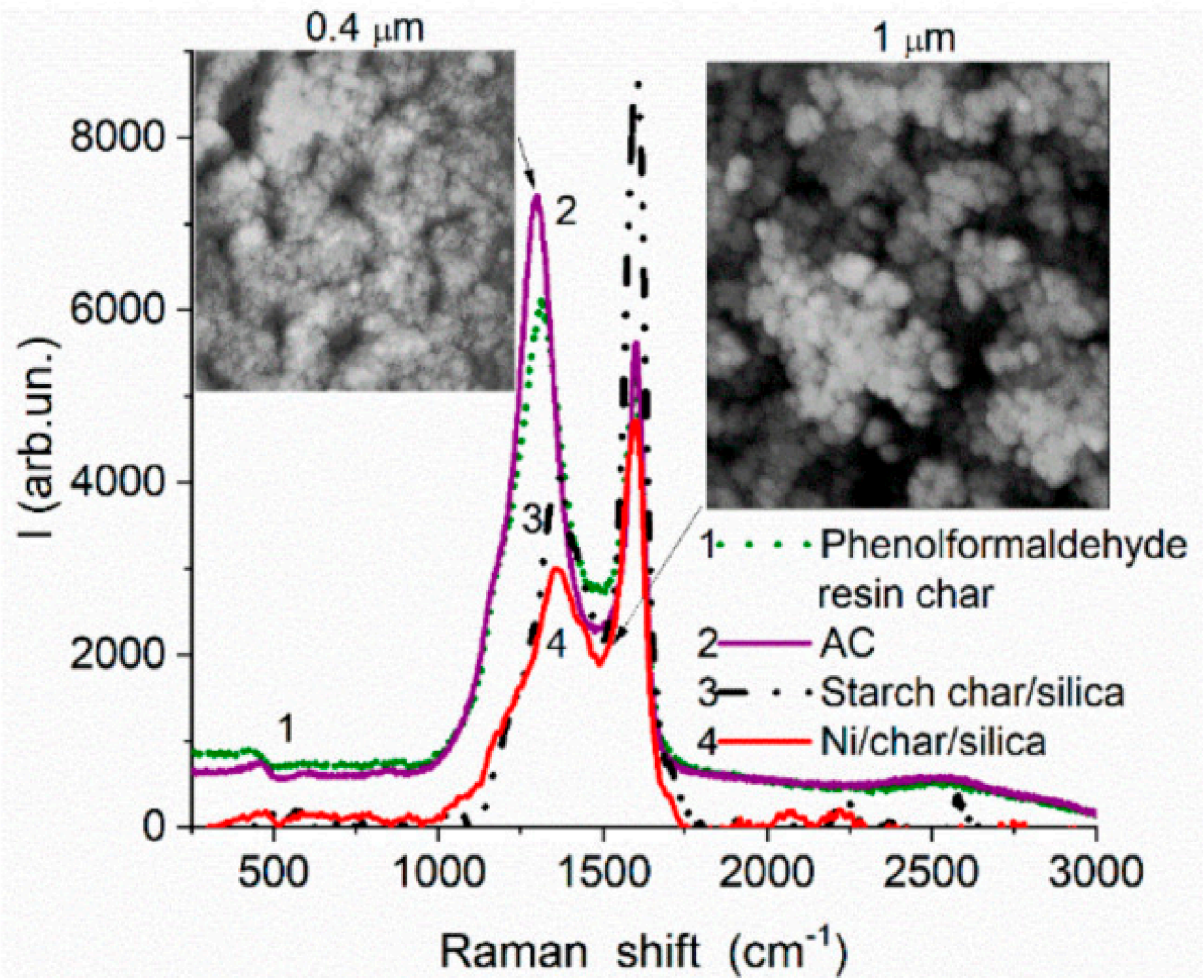
Typical Raman spectrum of phenolic resin carbonization products (Curve 1) [18].

The pyrolysis process primarily produces amorphous carbon [14], which exhibits a low degree of graphitization (as indicated by a high *I_D_/I_G_* ratio in the Raman spectrum). In the Raman spectroscopic analysis of phenolic resin carbon layers, the D band and G band are two key characteristic peaks used to evaluate the degree of microstructural order: The graphite band, located at approximately 1580–1600 cm^−1^, originates from the in-plane stretching vibrations of sp^2^-hybridized carbon atoms in the carbon rings and represents the degree of order in the graphite structure of the material, while the D peak (disorder band) is located at approximately 1340–1360 cm^−1^, arising from the breathing vibration mode of sp^2^-hybridized carbon atoms; its presence directly indicates disorder and defects in the carbon structure. In the study of phenolic resins, *I_D_*/*I_G_* is a key indicator of the degree of disorder in the carbon layer—a higher ratio indicates a lower proportion of relatively ordered graphite structure in the material, with disordered carbon (amorphous carbon) predominating [15,16], as shown in Figure 2. For traditional phenolic resins, the pyrolytic carbon layer typically exhibits a high *I_D_*/*I_G_* ratio, which directly confirms that the carbon layer consists primarily of amorphous carbon with low graphitization and a loose structure, thereby explaining why the carbon layer formed by traditional phenolic resins is brittle and prone to powdering and flaking [17]. It is worth noting that as the heat treatment temperature increases, the *I_D_*/*I_G_* ratio of phenolic resin char typically follows a pattern of first increasing and then decreasing, with a structural transition point occurring near approximately 700 °C. This actually shows that when the heat treatment temperature is higher, the carbon layer starts to transform into a more ordered structure. As a result, its interior becomes relatively loose and porous, with many small cracks and defects. Its inherent strength is relatively low, and it is particularly prone to breaking.

The structural integrity of the char layer is compromised through a combination of interrelated degradation mechanisms. First, there is a significant difference in the thermal expansion rates of organic and inorganic components upon heating, due to a mismatch in their thermal expansion coefficients. Thus, after a fire causes a sudden temperature spike that is followed by fast cooling, the rapid temperature change creates substantial internal stress. That stress often leaves the char, which is already quite brittle, full of tiny cracks called microcracks. Additionally, ongoing oxidation erosion slowly eats away at the char matrix. It turns parts of the char into gases and makes the pores bigger, which makes the char more likely to crack or break. At the same time, volatile gases from inside rush outward, putting a mechanical pull on the char. Sometimes, this lifts the char into blisters or peels it off in layers, especially where the char is thin or has flaws. In the end, even if the char survives these attacks, its poor stickiness to the original material underneath often becomes the main weak spot. This leads to the char completely detaching and turning into powder when there is any load on it. Recent tests at different scales have shown that this powdering process happens in four steps: interfacial separation, matrix cracking, delamination, and fiber breakage [19].

### 2.3. The Trade-Off Between “High Flame Retardancy” and “Low Charring” and Strategies for Achieving a Balance

Theoretically, improving flame retardancy is typically achieved by promoting char formation and increasing the thickness of the char layer. However, if the resulting char layer has low strength and poor adhesion, increasing its thickness may actually increase the risk of chalking. Therefore, the core scientific question and objective of this review is how to construct a char layer that is high-strength, highly oxidation-resistant, dense and continuous, and firmly bonded to the substrate.

A unified material design strategy should center on several complementary directions. The first step that needs to be taken is to make the carbon layer stronger from the inside. This can be achieved by increasing its graphitization degree and cross-linking density. Methods like catalytic graphitization or heteroatom-induced cross-linking can help with that [20]. At the same time, it is essential to ensure the interface between the carbon layer and the substrate remains stable. This can be achieved by strengthening chemical bonds or by adjusting the surface to improve mechanical interlocking. That way, when there is stress, delamination can be effectively prevented. Another key point is to build a multifunctional gradient protective layer. In this layer, the flame-retardant system turns on barrier, expansion, and ceramic-forming functions at different temperature stages. Eventually, this creates a stable shield that is graded in structure. Finally, consideration should also be given to reducing internal thermal stress. This can be accomplished through toughening modifications and the incorporation of flexible components. This helps the material handle thermal strain better and lowers the chance of cracks developing.

## 3. Research Progress on Key Technologies for High-Flame-Retardant, Low-Powdering Phenolic Boards

### 3.1. Chemical Strategies: Resin Molecular Design and Modification

#### 3.1.1. Elemental Modification

**Figure 3:**
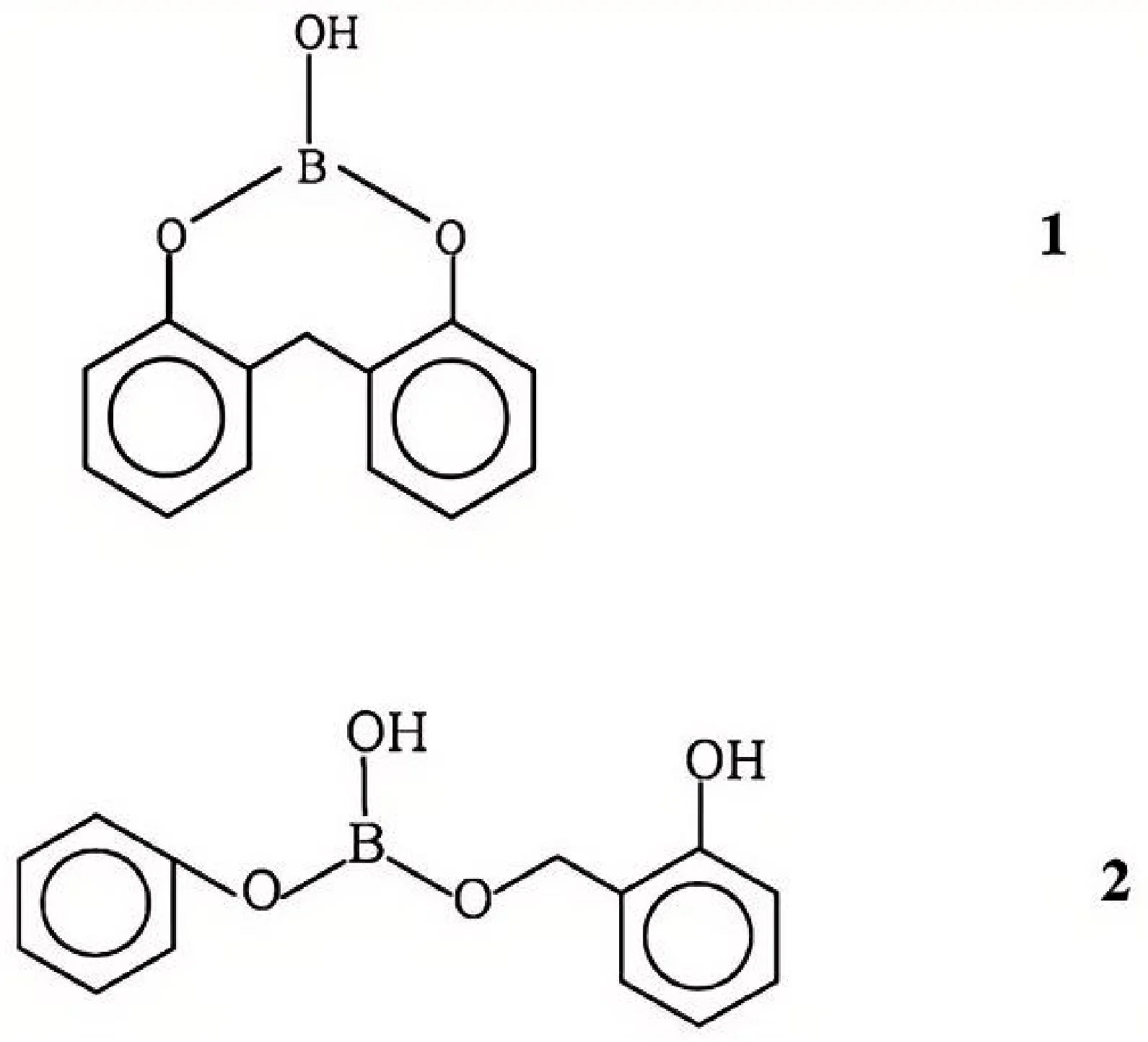
Formula of boron-modified resin. 1 (90 °C product): At this temperature, the reaction between triphenyl borate (TPB) and paraformaldehyde (PF) proceeds to a relatively low extent, resulting in a shallow crosslinked network. Consequently, the resin remains capable of melting and flowing upon heating. 2 (120/130 °C product): At higher temperatures, the reaction proceeds more thoroughly, forming a denser B–O–C crosslinked structure. After curing, the resin becomes insoluble and infusible, exhibiting higher thermal stability and char yield, but it cannot be reprocessed [22].

**Figure 4:**
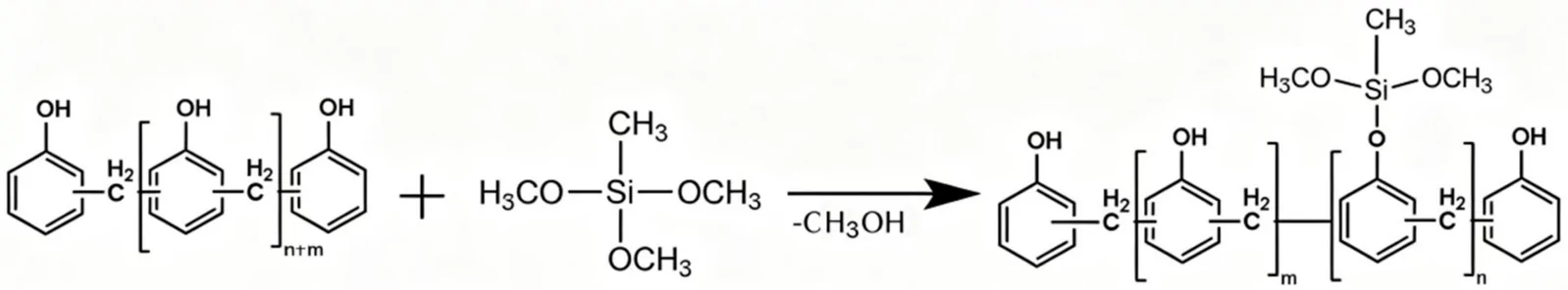
Molecular formula of silicone-modified resin [21].

Boron modification (Figure 3) has a fairly obvious drawback—it significantly reduces the toughness of the resin, making the material more brittle and less impact-resistant. Moreover, boron compounds may gradually hydrolyze when left in a humid environment for a long time, which can affect their long-term stability. Silicon modification has been reported as an effective approach to enhance the performance of phenolic resins. Essentially, it involves adding organosilicon units (such as phenyltrimethoxysilane or tetraethoxysilane) through copolymerization or grafting. This way, when combustion occurs, the silicon element moves to the surface of the material, forming a cross-linked network like Si-O-Si or Si-C, which eventually turns into a layer of silica or silicate ceramic. This ceramic layer is particularly heat-resistant and chemically stable, acting like a sturdy armor to protect the underlying carbon layer from oxidative erosion, thus effectively inhibiting the chalking phenomenon. Additionally, adding silicon can actually enhance the toughness of the resin [21] (Figure 4). However, silicon modification is not perfect either. It usually requires a very high thermal decomposition temperature (over 600 °C) to form a truly effective ceramic protective layer. But in real fire scenarios, the temperature rises extremely quickly, and this ceramic layer might not have enough time to fully form. Furthermore, these silicon-containing precursor materials themselves are relatively expensive, and if too much is added, it can actually make the resin difficult to process.

Phosphorus-based compounds mainly work in the condensed phase. They promote dehydration reactions, help form a carbon layer, and also create a coating of phosphate or polyphosphate. As for nitrogen-based compounds, they are mainly used as foaming agents. They produce inert gases like ammonia, which can dilute flammable gases and oxygen, while also providing flame retardant effects in the gas phase.

Among the B, Si, P and N modifications, boron modification is the most effective one for improving the quality of the char layer at present. This is proved by Raman spectrometry: the *I_D_*/*I_G_* shows a decrease from 2.63 to 1.32, a decrease of 49.8% [23,24,25,26], which means that it promotes graphitization significantly. However, B modification suffers from increased brittleness and potential hydrolytic instability in humid environments. Silicon modification offers superior toughness and ceramic layer formation but requires pyrolysis temperatures exceeding 600 °C for effective SiO_2_ vitrification—a condition that may not be met during rapid fire scenarios. Phosphorus modification is very good at promoting dehydration char formation at relatively low temperatures, which makes it effective in the early stages of a fire. But, phosphorus-containing compounds can volatilize at high temperatures, thus limiting long-term protection. Nitrogen modification is frequently used in combination with phosphorus, known as the P-N synergy, which enhances intumescence. However, nitrogen alone does not significantly contribute to char strength. Currently, no single-element modification effectively addresses the chalking problem. The most promising solution seems to be multi-element cooperative systems that leverage the complementary benefits of various elements. A summary of boron and silicon modification effects on char properties is presented in Table 1.

Boron-11 NMR spectroscopy of boron-modified phenolic resin reveals two principal signals. One is around 13 to 15 ppm, which corresponds to the triangular BO_3_ structure; the other is around 0 to 2 ppm, corresponding to the tetrahedral BO_4_ structure. Interestingly, after heat treatment, the ratio of BO_4_ to BO_3_ increases. This change in ratio is directly related to the carbon layer becoming more dense and performing better.

#### 3.1.2. Copolymer Modification

Regarding copolymer modification, the introduction of copolymerization or blending with resins with better thermal stability and charring efficiency, such as polyimide (PI), benzothiazole (BZ) and cyanate ester (CE), can improve the initial decomposition temperature, residual char yield and char layer quality of the phenolic resin matrix. For example, a phenolic/benzoxazine blend system can form an interpenetrating network structure, resulting in a denser char layer [31]. Blending with elastomers or thermoplastic polymers, like nitrile rubber (NBR), carboxyl-terminated nitrile rubber (CTBN) [32], and polyethersulfone (PES) [33], it forms an “island-sea structure” or toughening phase. This then causes silver streaks and shear bands, which release energy and reduce stress at crack tips. This improves matrix toughness, alleviates thermal stress concentration, and reduces the tendency of the carbon layer to crack. The key lies in controlling the particle size and distribution of the toughening phase, as well as its compatibility with the phenolic resin.

#### 3.1.3. Molecular Design and Hybrid Resin Systems

In addition to conventional modification methods, bio-based phenolic monomers such as resveratrol have been used in recent years to synthesize high-performance bio-based thermosetting resins due to their high phenolic hydroxyl density and rigid biphenyl structure. A resveratrol-based phenolic amine curing agent was synthesized via the Mannich reaction, which exhibited a carbonization temperature as high as 96.8 °C, demonstrating excellent carbonization capability and thermal stability, thereby offering a promising pathway for the bio-based replacement of phenolic resins [34]. Recent studies have further advanced biomass substitution toward completely formaldehyde-free systems. Fully bio-based phenolic foams completely free of phenol and formaldehyde were successfully fabricated by combining hydrothermally degraded lignin (via ethanol-induced hydrogenolysis) with condensed tannins [35]. Even with a lignin substitution ratio as high as 30 wt%, the foam maintained UL94 V-0 flame retardancy, and its peak heat release rate was comparable to that of pure tannin foam. The study indicates that moderate degradation of lignin (reduction in molecular weight) is a key prerequisite for achieving satisfactory foaming results. This provides a “zero petroleum-based” technical pathway for the green manufacturing of phenolic materials.

In the context of molecular design for hybrid resin systems, the bonding properties of poly-MDI-modified phenolic resin (PF/pMDI) used for the flame-retardant treatment of wood-based materials have been explored [36]. Findings revealed that flame-retardant impregnation significantly diminished the bonding strength of pure PF resin. But, adding pMDI substantially made up for the strength loss from the flame-retardant treatment, and the best amount to add turned out to be around 5% to 10%. So, this gives a new way to balance mechanical properties and flame retardancy in phenolic-based composites. Basically, it helps by combining complementary resin systems to reduce the bad effects that functional additives can have on mechanical performance.

### 3.2. Chemical Strategies: Advanced Flame-Retardant Systems and Cooperative Effects

It is difficult for a single flame retardant to effectively balance flame retardancy, mechanical properties, and resistance to chalking; therefore, the development of multi-component, multi-mechanism cooperative flame-retardant systems has become a leading research focus.

**Figure 5:**
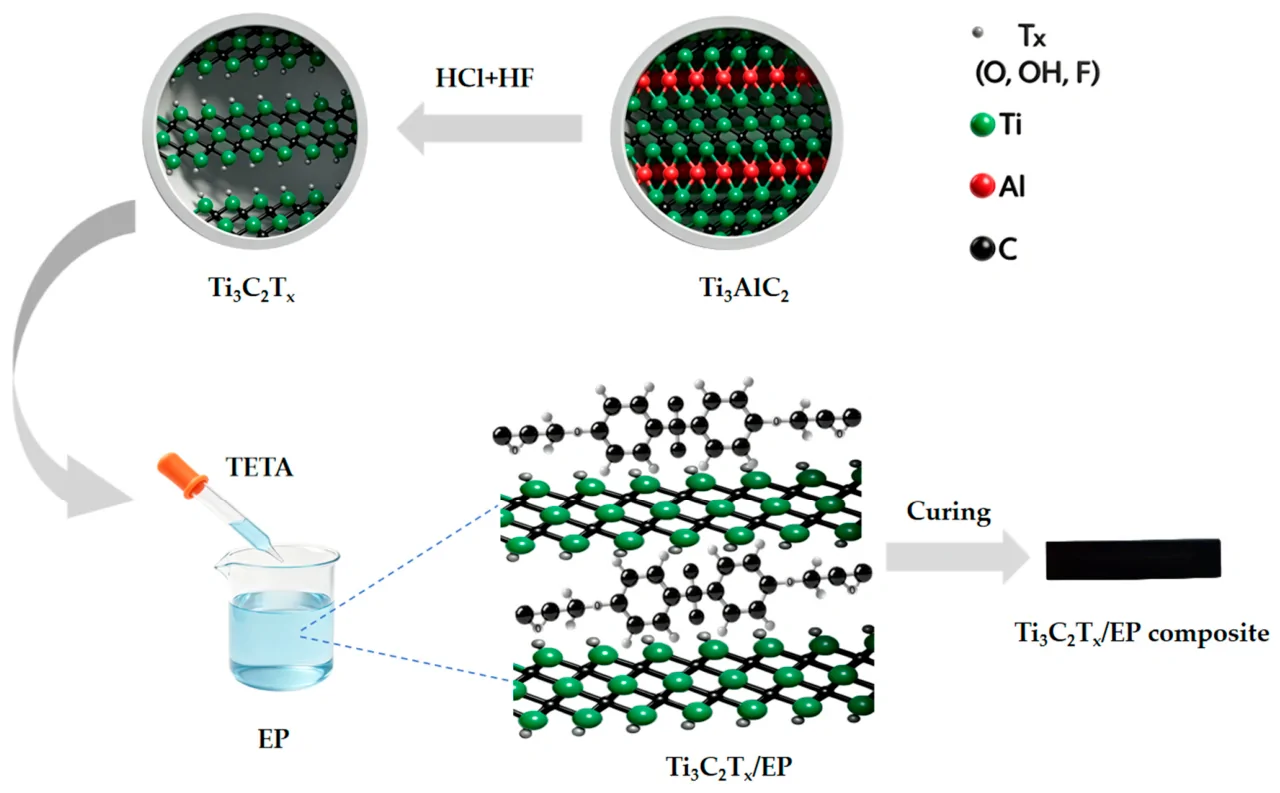
Two-dimensional transition metal carbides [42].

In terms of nanocomposite flame-retardant technology, layered silicates, such as organically modified montmorillonite (MMT), can exfoliate into nanoscale layers within the resin and disperse uniformly. During combustion, these layers migrate to the surface, forming a physical barrier that extends the escape path for pyrolysis gases and degradation products, while facilitating the development of a denser, higher-strength “brick-and-mortar” carbon layer. At the same time, the nanoscale layers also serve as reinforcing agents to enhance the stiffness of the carbon layer [37]; carbon nanomaterials (such as carbon nanotubes (CNTs), graphene, and their derivatives) possess extremely high specific surface areas, aspect ratios, and intrinsic mechanical strength. When incorporated into resins, they can form three-dimensional thermal and electrical networks or reinforcing frameworks within the resin [38]. During combustion, they not only catalyze the resin’s charring reaction but also act as reinforcing agents for the carbon layer, significantly enhancing its resistance to delamination and collapse. Furthermore, functionalized (e.g., hydroxylated, carboxylated) carbon nanomaterials can form stronger interfacial bonds with the resin matrix [39]. Two-dimensional transition metal carbides/nitrides (MXenes, such as Ti_3_C_2_N_x_) are emerging two-dimensional nanomaterials (Figure 5). Their surfaces are rich in functional groups (–OH, –F, etc.) that readily interact with resins, and their excellent electrical conductivity aids in dissipating static electricity and heat. At high temperatures, MXene may transform into metal oxides such as TiO_2_, combining with the carbon layer to form a robust ceramic/carbon composite protective layer that provides both physical barrier and catalytic carbonization effects [40,41]. Between 2024 and 2026, research on MXene in the field of polymer flame retardancy will shift its focus toward composite applications with phase-change materials. MXene/graphene composite phase-change materials have been utilized for thermal management in lithium-ion batteries, demonstrating excellent thermal stability in 3s and 3p battery modules [42]. This strategy can be extended to the development of fire-retardant materials for phenolic-based batteries.

**Figure 6:**
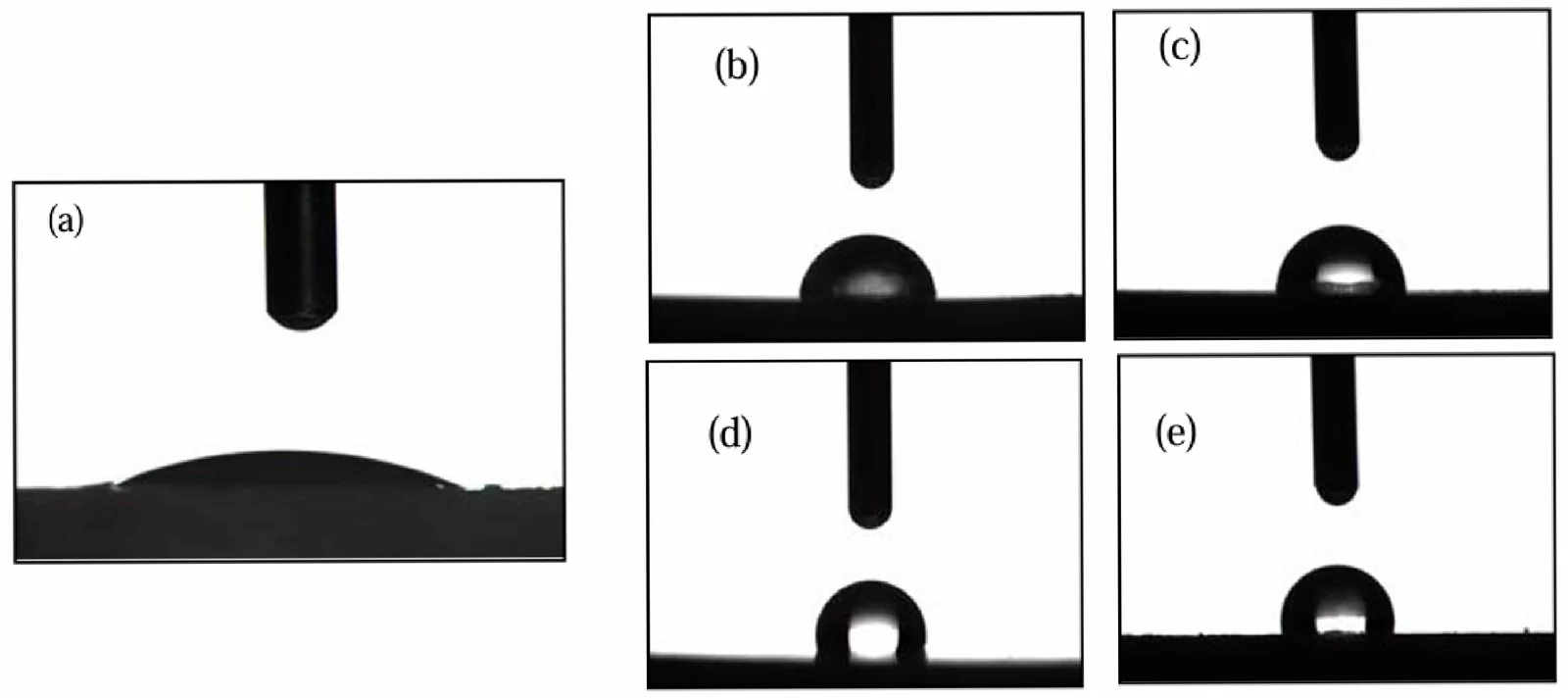
Water contact angle of tung oil-modified phenolic resin [45]. WCA photographs: (**a**) APP, (**b**) HTPFAPP, (**c**) ETPFAPP, (**d**) Heat cured HTPFAPP, (**e**) Heat cured ETPFAPP.

Regarding the application and optimization of intumescent flame retardant (IFR) systems, traditional IFR systems typically consist of an acid source (such as ammonium polyphosphate, APP), a carbon source (such as pentaerythritol, PER), and a gas source (such as melamine, MEL) [43]. When incorporated into phenolic resin systems, these components form an intumescent char layer on the material’s surface upon heating, with an expansion ratio reaching tens of times the original volume. This char layer is low in density and high in porosity, providing excellent thermal insulation. Furthermore, due to its intumescent properties, it effectively encapsulates the substrate, offering superior resistance to cracking and chalking compared to traditional dense but brittle char layers. Current research focuses on (1) improving the compatibility of IFR with phenolic resins through microencapsulation and surface modification (e.g., treating APP with phosphorus-containing silane coupling agents [44]) to prevent migration and leaching; for example, in situ polymerization of tung oil-modified phenolic resin to encapsulate APP can significantly increase its water contact angle from 23° to 106–114° (Figure 6), effectively enhancing hydrophobicity and interfacial compatibility; (2) developing new, highly efficient IFRs, such as triazine-based carbonizers, bio-based carbon sources (starch-based, lignin, etc.), and synergists with carbonization catalytic effects (e.g., zeolites, metal oxides), as well as blend IFRs with small amounts of nanomaterials (e.g., modified MMT, CNTs) to utilize the nanomaterials to improve the strength, toughness, and density of the expanded carbon layer.

In the field of bio-based and sustainable flame retardants, natural biomass resources such as phytic acid, chitin, lignin, cashew nut shell liquid, and DNA are used as flame retardants or synergists. These substances are rich in flame-retardant elements such as phosphorus, nitrogen, and carbon, and possess inherent charring properties. These structures have multiple hydroxyl groups and active sites. This helps them form strong hydrogen bonds or chemical bonds with phenolic resins. So, the interface becomes better, and the char layer’s adhesion improves [46]. Actually, this fits well with the principles of green chemistry and sustainable development. Recent studies have further expanded the scope of bio-based flame retardants. A novel starch-based flame retardant was prepared using sodium tripolyphosphate-modified starch. When blended with phenolic resin, it reduced the powdering rate of the composite material by 28.42%, increased the char yield by 42.71%, and passed the UL94 V-0 rating test [45]. Modification with fly ash-derived geopolymers can induce a transition in phenolic foam from an open-cell to a closed-cell structure, reducing the charring rate by 41% [47]. A systematic review of molecular engineering strategies for recyclable bio-based thermosetting resins has highlighted that the introduction of dynamic covalent networks can integrate recyclability and flame retardancy in phenolic resins [48].

Using nanocomposites is the most comprehensive way to enhance performance. Those nanofillers can boost several properties all at once, such as barrier performance, the strength of the char layer, and smoke suppression. However, achieving nanoscale uniform dispersion on an industrial scale remains a major challenge. Moreover, materials such as functionalized carbon nanotubes, graphene, and MXenes are considerably more expensive than traditional additives. The IFR system expands upon heating, providing excellent thermal insulation. However, its compatibility with the PF matrix requires precise formulation adjustment. Microencapsulation effectively addresses the migration issue, yet it introduces additional processing complexity. Bio-based flame retardants represent an environmentally sustainable direction; nevertheless, their flame retardant efficiency and long-term stability currently fall short of those of synthetic alternatives. In fact, the most effective strategy reported in the literature involves combining nanocomposites with intumescent systems, thereby simultaneously improving char layer quality and fire resistance, reinforcing both performance aspects [39,49]. The flame retardant systems for phenolic resin composites are summarized in Table 2.

### 3.3. Physical Strategies: Fiber–Matrix Interface Design

Improving the fiber–resin interface is extremely important. It helps keep composite materials strong and stable when it is hot, and makes sure they do not delaminate. Therefore, systematic optimization should be achieved through two primary strategies: fiber surface modification and multi-scale hybrid reinforcement. The carbon fiber-reinforced system showed the best mechanical properties—its tensile strength was 54.62 MPa and flexural strength 29.69 MPa. It also had excellent ablation resistance, with a linear ablation rate of 0.13 mm/s. This renders it highly suitable for high-load or extreme ablation environments [50]. Meanwhile, the quartz fiber-reinforced system had the lowest thermal conductivity, just 0.1 W/(m·K), so it works well in high thermal insulation environments. For surface treatment, chemical coupling agents like silanes or titanates can be used on glass or basalt fibers. These add reactive groups, such as amino or epoxy groups, that react with the resin to form a strong chemically bonded interface layer [51]. Another option is low-temperature plasma treatment. It etches and activates the fiber surface, introducing polar functional groups. This significantly enhances the surface energy and improves how well the resin wets the surface [52]. Additionally, a sizing agent with good resin compatibility and high-temperature resistance—like polyurethane or epoxy modifiers—can be coated onto the fiber surface, or alternatively, nanomaterials such as nano-SiO_2_ and carbon nanotubes to build a tough interfacial transition layer [53].

It is worth noting that the nanoporous structure of phenolic aerogels weakens the bond strength at the fiber–resin interface, which is a key bottleneck limiting the improvement of mechanical properties and must be overcome through interface engineering (such as nano-coating of fiber surfaces). Micro–nano hybrid reinforcement employs a multi-scale composite model where “micron-scale continuous fibers (such as glass fiber fabric) bear the primary load, while nanoscale particles (such as CNTs and nanoclays) reinforce the interface and matrix”. Nanoparticles can fill microdefects in the interface region, form physical interlocking, and bridge microcracks, thereby significantly enhancing the residual strength of the interface at high temperatures [54]. Fiber hybrid reinforcement, on the other hand, involves blending fibers of different types and moduli (e.g., carbon fibers with glass fibers or aramid fibers with glass fibers). By leveraging their differing coefficients of thermal expansion and failure modes, this approach achieves a more uniform stress distribution, suppresses the rapid propagation of single cracks, and thereby effectively improves the composite material’s overall resistance to powdering [55].

### 3.4. Physical Strategies: Molding Process Optimization and Process Control

Molding process parameters critically influence anti-chalking performance by determining internal structure uniformity and defect content. Low-pressure molding processes, including RTM (0.4–0.7 MPa, four-stage pressure control), VARI (vacuum-assisted, approximately 0.1 MPa), and Light RTM, involve different trade-offs among surface quality, dimensional accuracy and cost. Uniform fiber wetting and controlled curing are critical for preventing chalking. These factors, guided by DSC and rheological analysis, reduce internal stress concentrations that can initiate cracks. Precise control over blowing agent decomposition and post-curing protocols further enhances performance [56]. Post-curing, conducted at temperatures slightly above service temperature, increases cross-linking density and eliminates residual stresses. As a result, char layer cohesion improves, and resistance to powdering is directly enhanced [57,58,59]. The choice of molding technique—whether RTM, VARI, or Light RTM—directly shapes this internal quality through distinct pressure profiles and flow dynamics.

**Figure 7:**
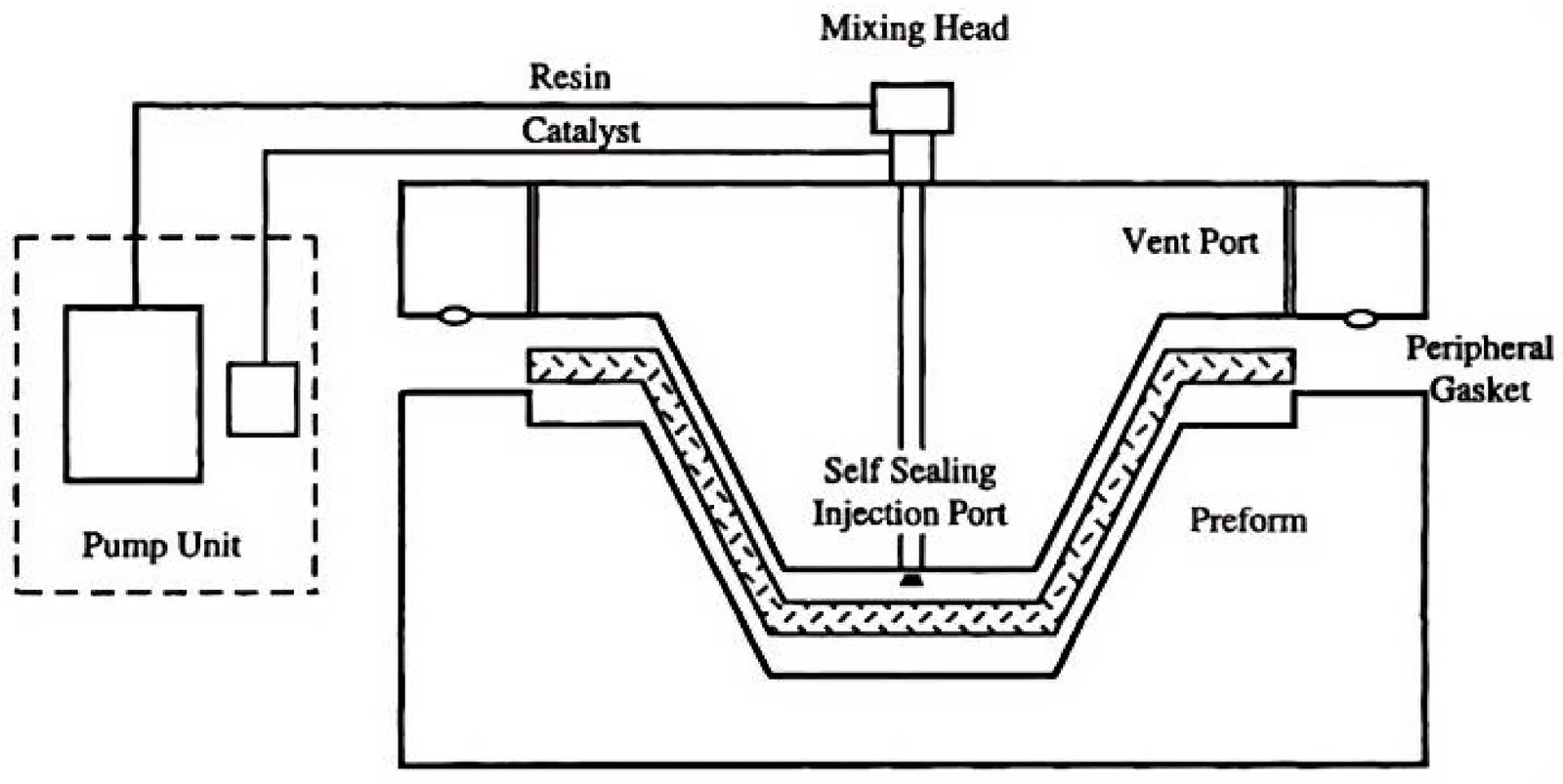
Schematic of the RTM process [60].

**Figure 8:**
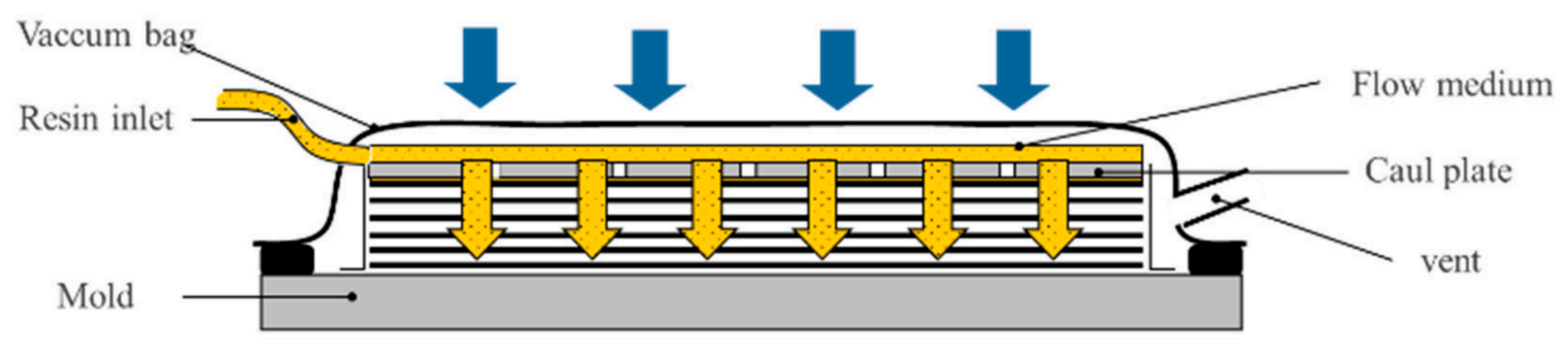
VARI process [63].

(1) RTM process: Using a double-sided rigid mold, the RTM process flowchart is shown in Figure 7. Dry fiber-reinforced material is placed on the lower mold, and the molds are closed and clamped. Low-viscosity resin is injected into the closed mold cavity under an external pressure of 0.4–0.7 MPa. After the fibers are impregnated, the part is cured and demolded. A four-stage pressure control strategy (0.2 → 0.3 → 0.4 → 0.5 MPa) can effectively improve the wetting uniformity of the fiber preform, reducing defects such as dry spots and voids. Its advantages include smooth surfaces on both sides of the product, high dimensional accuracy, controllable fiber content, and low VOC emissions, making it suitable for manufacturing complex components with high requirements, such as aerospace structural parts and automotive components. Limitations include the relatively high cost of double-sided molds and challenges in achieving complete resin impregnation for extra-large or extremely thick components. In RTM molding of phenolic aerogel composites, this refined pressure control method can be extended to low-pressure molding processes for high-performance phenolic panels.

(2) The VARI process employs a single-sided rigid mold in conjunction with a vacuum bag. A vacuum pump is used here to create a negative pressure environment inside the mold cavity. Atmospheric pressure—approximately 0.1 MPa—then draws the resin through pre-placed tubes, allowing it to infiltrate the fiber preform [61,62]. A schematic diagram of the RTM process is shown in Figure 8 (Blue arrows indicate the direction of cooling water circulation through the cool plate. Yellow arrows indicate the flow path of the resin medium.) Its prominent advantages include mold costs that are a fraction of those for RTM, virtually unlimited dimensions (capable of producing wind turbine blades or ship hulls tens of meters long), and the vacuum compaction effect, which helps achieve parts with high fiber content and low porosity. There are two main limitations. First, only the surface in contact with the mold is smooth; the other side requires subsequent finishing. Second, resin flow is influenced by multiple factors, including vacuum level, resin viscosity, ambient temperature, and layup structure. This makes process control relatively complex and can lead to dry spots or uneven impregnation.

(3) Light RTM is like a middle ground between the first two methods. It uses a semi-rigid mold where the lower half is rigid and the upper half is flexible. Actually, the lower mold is rigid, while the upper mold is made of composite material or thin metal, just a few millimeters thick. It works with a two-stage vacuum system. First, a high-pressure vacuum seals the mold. Then, a low-pressure vacuum helps the resin flow. The injection pressure is usually under 0.1 MPa. So, this process brings together the smooth surfaces from RTM and the cost-saving benefit of VARI. The parts exhibit good surface quality on both sides, with mold investment and part precision falling between those of the first two methods. It is suitable for medium-volume production of up to approximately 20,000 units per year, such as yacht components and wind turbine nacelle covers. These molded panels can actually be further post-cured placing them in an environment slightly higher than their normal operating temperature for an extended period, a process known as “post-curing”. This helps to make the cross-linking within the resin more compact, releasing internal residual stress, thereby increasing its glass transition temperature (TG) and thermal stability [59].

## 4. Performance Evaluation

### 4.1. Characterization of Flame Retardant Properties

The limiting oxygen index (LOI) test, conducted according to [64] or [65], determines the minimum oxygen concentration in a nitrogen-oxygen mixture required for a material to sustain combustion. This value serves as a fundamental indicator of a material’s flammability. For applications demanding high flame retardancy, an LOI value exceeding 40% is typically targeted [49].

The vertical and horizontal combustion test, formally titled the Standard for Safety of Flammability of Plastic Materials for Parts in Devices and Appliances, is commonly employed to assess the combustion behavior of materials under small flame exposure [66]. This test records several key indicators, including the self-extinguishing time following flame application, whether molten droplets are generated during combustion, and whether any such droplets ignite the underlying degreased cotton. Among the classification levels, V-0 represents a highly stringent flame retardancy rating. To achieve this rating, the total self-extinguishing time after two separate 10 s ignitions must not exceed 10 s, and no flaming droplet that ignites the cotton wool is permitted.

The fire performance of building materials is classified according to GB 8624 [3]. Test methods include the single burning item (SBI) test and the non-combustibility test [67]. Based on the results, the material is rated as either Class A2 (non-combustible) or Class B1 (difficult to ignite). This rating reflects the material’s overall fire behavior under realistic fire conditions. These tests enable a rapid assessment of a material’s basic flame-retardant rating, providing a basis for preliminary material screening and matching with specific application scenarios.

**Figure 9:**
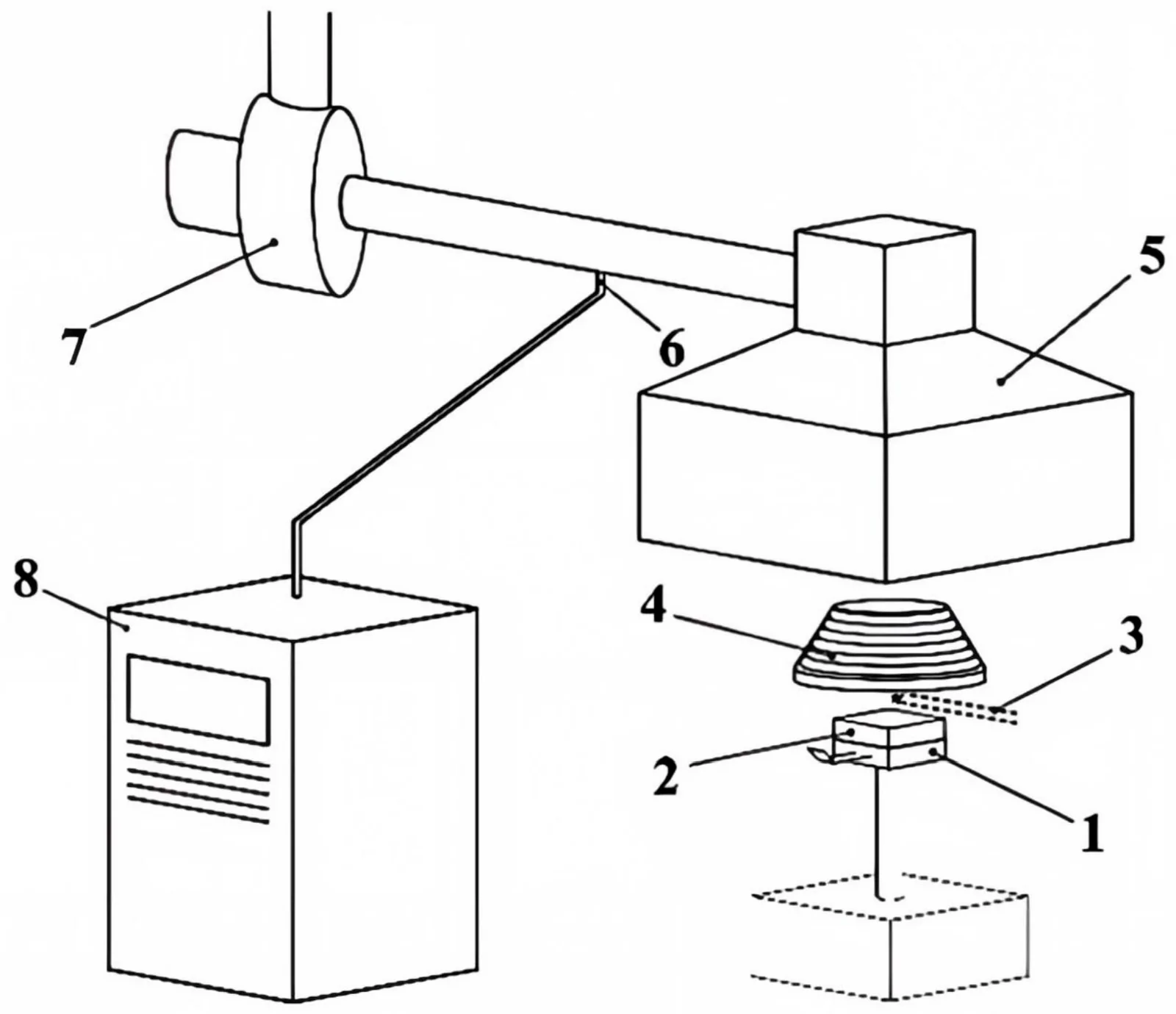
The schematic of the cone calorimeter: (1) sample holder, (2) sample, (3) igniter, (4) cone heater, (5) exhaust hood, (6) combustion gas sample extraction, (7) fan, (8) combustion gas analyzer [69].

A cone calorimeter is used to simulate real-world fire conditions; a schematic diagram of the cone calorimeter is shown in Figure 9, and it enables a comprehensive assessment of a material’s combustion behavior [68]. This test yields several key parameters: the heat release rate (HRR) and its peak value (pHRR) are important indicators of the rate of fire development; a lower pHRR value indicates that the material releases less heat during combustion, resulting in slower fire spread; the total heat release (THR) reflects the total heat released by the material throughout the combustion process; a lower THR indicates that less total energy is released during combustion; effective heat of combustion (EHC) is used to assess the completeness of gas-phase combustion; a lower EHC typically indicates that flame retardants have exerted an inhibitory effect in the gas phase; a lower mass loss rate (MLR) indicates a slower pyrolysis rate of the material and more effective char layer protection, which also indirectly reflects the material’s ability to resist charring and structural damage. In addition, the smoke production rate (SPR) and total smoke production (TSP) are used to evaluate a material’s smoke-generating properties, while the specific extinction area (SEA) reflects the light-blocking capacity of smoke particles. The production rates of carbon monoxide (CO) and carbon dioxide (CO_2_) are key parameters for assessing combustion toxicity. Cone calorimeter testing comprehensively reveals a material’s heat release, smoke generation, and toxic product release behavior during a fire, making it a core method for comprehensively evaluating a material’s fire safety.

Thermogravimetric analysis (TGA) is conducted in nitrogen and air environments to assess a material’s thermal decomposition temperatures. The initial decomposition temperature (T_5%_) is defined as the temperature at which a 5% mass loss is observed. T_max_ denotes the temperature at which the rate of mass loss in the sample reaches its peak value. Furthermore, TGA offers insights into weight loss rates at different stages and the quantity of carbon residue remaining after high-temperature exposure. A high carbon residue is direct evidence that the condensed-phase flame-retardant mode of action is functioning effectively, indicating that the material can form a dense carbon layer at high temperatures, thereby blocking the further transfer of heat and oxygen. TGA can quantitatively evaluate a material’s thermal stability, decomposition behavior, and carbonization capability; a high carbon residue is an important indirect indicator of a material’s resistance to charring.

Differential scanning calorimetry (DSC) is employed to obtain thermal parameters, for example, the glass transition temperature (TG) and the heat of curing. In addition, it reveals how the material’s physical state and chemical reactivity evolve during heating. Together, these measurements facilitate a thorough evaluation of thermal stability and temperature resistance.

### 4.2. Evaluation of Anti-Powdering Performance

Existing methods for evaluating anti-powdering performance primarily include the vibration method, the airflow sweeping method/fluidized bed method, the rotary wear method (drum method), and the falling hammer/impact crushing method. These methods differ significantly in terms of core principles, applicable scenarios, evaluation metrics, data characteristics, causes of data discrepancies, and the degree of standardization.

The vibration method is based on the mechanical reciprocating vibration, and completes the test by using the collision, friction and compression between particles. It is suitable for catalyst particles and pellets in transport, loading and mild fluidization. The main evaluation indices are pulverization rate, increment rate of fine powder and strength retention rate. The method is characterized by good data repeatability, moderate values and correspondence to the actual storage and transportation conditions and high degree of standardization. The data fluctuations are mainly due to the low-frequency collision force mechanism, and the absence of high-speed scouring leads to lower levels of powdering than the airflow sweeping method.

The airflow sweeping method/fluidized bed method relies on high-speed airflow scouring, entrainment, and high-speed particle collisions to conduct testing. It is suitable for porous/brittle particles under fluidized catalytic, adsorbent, and pneumatic conveying conditions. Key evaluation indicators include wear rate, pulverization rate, and particle size decay curves. Pulverization values are usually high and depend on the strength of the surface. The method is greatly affected by the speed of the gas and the structure of the bed, but there is a moderate level of standardization. The force characteristics of high-speed shear and gas flow impact result in significant differences from data obtained by the vibration method.

The rotary wear method (drum method) involves testing through particle tumbling and friction driven by drum rotation. It is suitable for larger-particle materials such as metallurgical pellets and ores, with a focus on overall wear resistance. Key evaluation indicators include the wear index and abrasion resistance index. The data exhibit strong stability. However, the continuous tumbling friction mechanism differs from the vibration-impact mode, making direct conversion of results with other methods impossible. This method has a high degree of standardization, and some aspects have already been established as industry standards.

The falling hammer/impact crushing method centers on applying pressure through a hammer dropped from a fixed height. It is suitable for high-strength structural materials such as briquettes and formed particles, focusing on the inherent strength of the particles. Key evaluation metrics include the crushing rate and the retention rate of compressive strength. The data exhibit significant variability. The stress mechanism of instantaneous impact loads is entirely different from the pulverization mechanisms associated with friction and abrasion. This method has a high degree of standardization and is often used for supplementary verification of strength.

Currently, the level of standardization varies significantly across these methods, and there is a lack of unified test parameters and evaluation criteria. This is a key issue of concern in the journal literature. This situation prevents effective comparison of experimental data across different research teams and makes it difficult to mutually recognize results, thereby hindering the standardized development of research on anti-powdering performance.

### 4.3. Comprehensive Performance Evaluation

As a complex composite material system, high-performance phenolic panels must strike a balance among various properties; therefore, a comprehensive evaluation system should cover their overall performance across multiple dimensions.

In terms of physical properties, attention should be paid to fundamental parameters such as density, thermal conductivity, water absorption, and dimensional stability. Regarding mechanical properties, key indicators such as flexural strength, tensile strength, compressive strength, impact toughness, and hardness must be systematically evaluated to ensure that flame-retardant and anti-chalking modifications do not significantly compromise the material’s mechanical load-bearing capacity. When checking how well phenolic resin coatings can handle humid and hot weather, the focus is on their resistance to conditions like humid-heat aging, freeze–thaw cycles, and UV aging [70]. The goal is to determine how their performance might degrade with long-term use.

In addition, processability directly determines the workability of the material and the quality of the finished part, as reflected in four specific aspects: resin viscosity, pot life, curing characteristics, and wetting of the reinforcing material. To control resin viscosity, an epoxy-rubber wet lay-up system was developed to significantly improve wetting of the fiber reinforcement by controlling the viscosity at 0.95 Pa·s, thereby avoiding dry spots and porosity defects [71]. A study in Colloids and Surfaces A (2018) [72] investigated pot life. It found that amine curing agents with shorter pot lives exhibit stronger C–N bond characteristic peaks. Pot life and curing rate are in a competitive relationship. If the pot life is excessively short, the resin may gel before complete wetting occurs. This leads to poor wetting. Dynamic mechanical analysis (DMA) and differential scanning calorimetry (DSC) have been employed to study the curing and post-curing behavior of vinyl ester resins, successfully determining gelation time and gelation temperature [73]. Meanwhile, the significant influence of montmorillonite nanofillers on the gelation time and curing kinetics of unsaturated polyester resins has also been revealed [74]. Regarding the wettability of reinforcing materials, researchers [72] determined surface tension using the pendant drop method and calculated the adhesion energy. By calculating the interfacial shear strength using the Drzal equation, they established a quantitative relationship between the wetting rate and the interlaminar shear strength in composite materials. Additionally, a review (2025) [75] clearly pointed out that physical or chemical modifications of phenolic resins can effectively improve their wettability toward fiber reinforcements. Viscosity, pot life, curing behavior, and wettability are coupled process parameters that collectively dictate the resin’s processing window. To ensure defect-free molding and satisfactory mechanical performance, these four factors must be jointly optimized, with attention paid to both resin formulation and curing cycle design.

Environmental and safety performance evaluations are essential and must include analysis of toxic combustion gases, smoke density testing, and life cycle assessment (LCA) to comprehensively evaluate the material’s environmental sustainability and fire safety. Only through the cooperative optimization and systematic evaluation of these multiple parameters can high-performance phenolic panels be developed that truly meet the demands of high-end applications.

It should be acknowledged that while the framework formally addresses a broad range of factors (e.g., physical, mechanical, weatherability, processing, and environmental safety), in practice, it serves less as a decision-support system and more as a compliance checklist. The core limitation is that it specifies which parameters ought to be measured, yet offers no guidance on how to act upon the measured results. In practice, trade-offs among conflicting properties are unavoidable, but the appropriate balance can only be determined by the specific demands of each application. In the absence of scenario-based weighting and clearly defined minimum performance thresholds, such an evaluation approach risks becoming a formal exercise that covers every aspect while guiding no practical decisions.

A more fundamental issue is that all the metrics currently employed are static values recorded at discrete points in time. The actual behavior of phenolic materials under fire conditions, however, unfolds as a continuous and dynamic process. Mechanical degradation, changes in thermophysical properties, and the growth of the char layer all exhibit strong time dependence. Single point measurements are incapable of capturing these evolutionary trends, let alone predicting the onset of sudden failure under extreme conditions. Moreover, weather resistance and fire resistance are currently assessed in separate tests, even though aging-induced surface microcracks and chemical deterioration may act as latent triggers for abrupt performance loss once a fire occurs. Processing characteristics are likewise treated as an isolated category, without establishing quantitative correlations with the final service performance of the finished components.

Perhaps the most critical weakness is the absence of any meaningful connection between this evaluation framework and the strategic approaches discussed earlier, such as multi-scale simulation and machine learning-based optimization. Evaluation ought to serve as the feedback loop that connects testing with subsequent iteration, not as a detached final inspection conducted at the end of the development chain. In summary, the current framework is comprehensive but limited in practical value. To guide materials innovation effectively, it must evolve. An application-oriented model is needed—one that emphasizes dynamic characterization and closed-loop data integration.

## 5. Application Prospects, Challenges, and Future Directions

### 5.1. Prospects for Emerging Application Areas

In recent years, artificial intelligence (AI) has been increasingly applied in flame-retardant material design, offering a new paradigm to overcome the efficiency bottlenecks of traditional trial-and-error approaches. AI-assisted molecular design and performance prediction have demonstrated significant efficacy in flame-retardant polylactic acid [76], epoxy resins for battery thermal protection [77], and polymer composites [78,79], driving the transition from “experience-driven” to “data-driven” development. Generative AI and molecular design algorithms enable rapid screening of high-performance flame retardants, substantially shortening R&D cycles [80]. This paradigm holds considerable promise for the development of high-flame-retardant, low-powdering phenolic boards.

**Figure 10:**
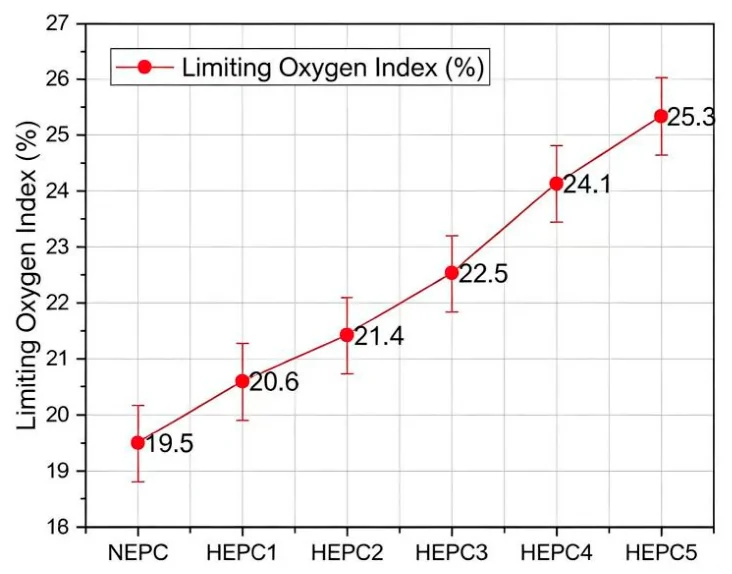
Limiting oxygen index values for mixed configurations [81].

In new energy applications, phenolic-based fireproof materials have demonstrated significant advantages in thermal runaway protection for electrochemical energy storage stations and electric vehicle battery packs, owing to their excellent flame erosion resistance, dimensional stability at high temperatures, and low thermal conductivity. APP-reinforced glass/phenolic and MPP-filled S-glass/phenolic composites have been confirmed to effectively enhance battery casing flame retardancy, achieving a UL94 V-0 rating at 12–15 wt% MPP loading [81,82] with their LOI values summarized in Figure 10. Commercial phenolic sheet molding compound (SMC) for EV battery boxes has been reported to achieve over 65% mechanical property retention after fire testing, with strength and impact energy absorption surpassing those of cast aluminum [83]. Furthermore, another study has reported 85% recycled/bio-based resin content and char strength exceeding 200 N after 30 min combustion [84]. In terms of industrialization, Teijin Automotive has successfully applied aviation-derived phenolic materials to EV battery enclosures, enabling non-melting and non-burning performance at high temperatures. Sumitomo Bakelite’s PM-5820 phenolic molding compound maintains structural integrity under 10 min blowtorch exposure, with thermal strength over 40 times the [66] and excellent thin-wall injection moldability, overcoming the shape limitations of conventional SMC and mica sheets.

In the aerospace and high-end transportation sectors, phenolic-based composites have become the material of choice for cabin structures and interior trim due to their lightweight properties, high specific strength, excellent flame-retardant and low-smoke characteristics, and resistance to chalking.

**Figure 11:**
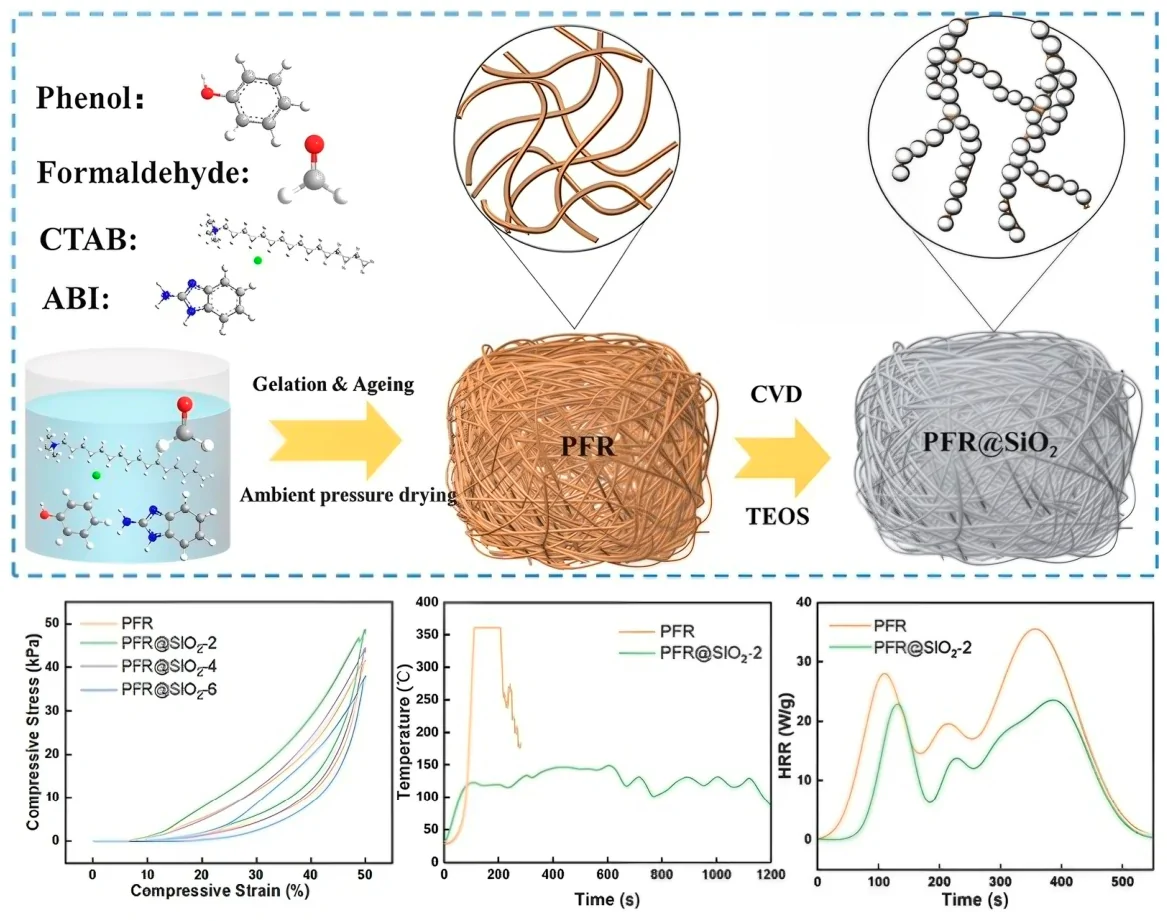
The preparation process, microstructure, mechanical properties, and fire-retardant properties of “PFR@SiO_2_ aerogel” [86].

For aerospace applications, the AeroFEATHER^TM^ honeycomb panel developed by Collins Aerospace features a sandwich structure combining phenolic fiberglass skin with an aramid fiber honeycomb core (Figure 11). Compared to traditional two-layer panels, it achieves a 38% weight reduction, while delivering a 300% increase in impact strength and an 8% increase in peel strength. It also meets the stringent flame-retardant requirements of [85], including the 60 s vertical burn test, the OSU heat release test, and the NBS smoke density test. U.S. Patent US5337288 (1992) addresses vibration and acoustic control. It discloses a sound and vibration damping composite material. The material consists of two layers. One is a constraint layer made of graphite fabric/phenolic resin composite. The other is a rubber-based viscoelastic layer. This design is specifically intended for vibration and noise reduction in aircraft, ships, and submarines. This material achieves excellent damping performance without adding weight, while also meeting flame-retardant requirements. Notably, in January 2026, Sumitomo Bakelite Co., Ltd. announced the development of the world’s first biomass-based PFA (furfural resin) prepreg, specifically designed for aircraft interior materials. Made from non-food biomass feedstocks, this product reduces the carbon footprint by 43% compared to traditional phenolic prepregs. It meets the stringent FST (flame-retardant, low-smoke, low-toxicity) standards of [85] and demonstrates performance comparable to that of petroleum-based phenolic resins in the OSU heat release test under 14 CFR Part 25. Mass production is scheduled to begin in 2028. This breakthrough marks the entry of bio-based flame-retardant materials into the engineering application phase in the aerospace sector.

Phenolic composites are key materials for high-speed rail interiors due to their light weight, high strength, flame retardancy, low smoke, sound insulation, and vibration resistance. The ETR500 train (Italy, 2004) uses phenolic fiberglass/Nomex honeycomb panels for walls, ceilings, and luggage compartments. These panels combine mechanical strength, light weight, flexural resistance, self-extinguishing, low heat release, and low emissions. Milwaukee Composites has developed flame-retardant phenolic panels (US 6,013,344; US 6,863,775 B2; US 7,618,514 B2) with a phenolic frame encapsulating the core. Designed for subways, high-speed trains, railcars, and buses, they eliminate steel underframes and insulation, reduce weight, meet fire standards, and offer better thermal/acoustic insulation than metal-clad plywood. Composite floor panels have been evaluated as a replacement for traditional plywood flooring in railway passenger cars, demonstrating that phenolic-based composites exhibit excellent fire resistance and structural integrity. In the fields of super-high-rise buildings and large-scale public facilities, phenolic-based composite materials have become the ideal choice for critical fire protection and structural reinforcement due to their exceptional fire resistance, low smoke and low toxicity, lightweight properties, and, crucially, low dust generation [87]. As modern buildings continue to grow in height and structural complexity, increasingly stringent requirements are being placed on the fire resistance ratings of building components. According to internationally accepted standards, the fire resistance rating of critical components in super-high-rise buildings, such as load-bearing columns and core walls, is typically required to reach 3–4 h, while the fire resistance rating of shaft walls in cable shafts and utility shafts must also reach 2 h. These heightened requirements stem from the fact that fires in super-high-rise buildings often burn for extended periods and are difficult to extinguish; if vertical shafts and other passageways are compromised, the fire can spread rapidly throughout the building. It standards clearly specify the test procedures for a 4 h fire resistance rating by [88]. During the test, the specimen must withstand a temperature rise from an initial 537 °C to 1093 °C over a 4 h period, while maintaining structural integrity and thermal insulation.

**Figure 12:**
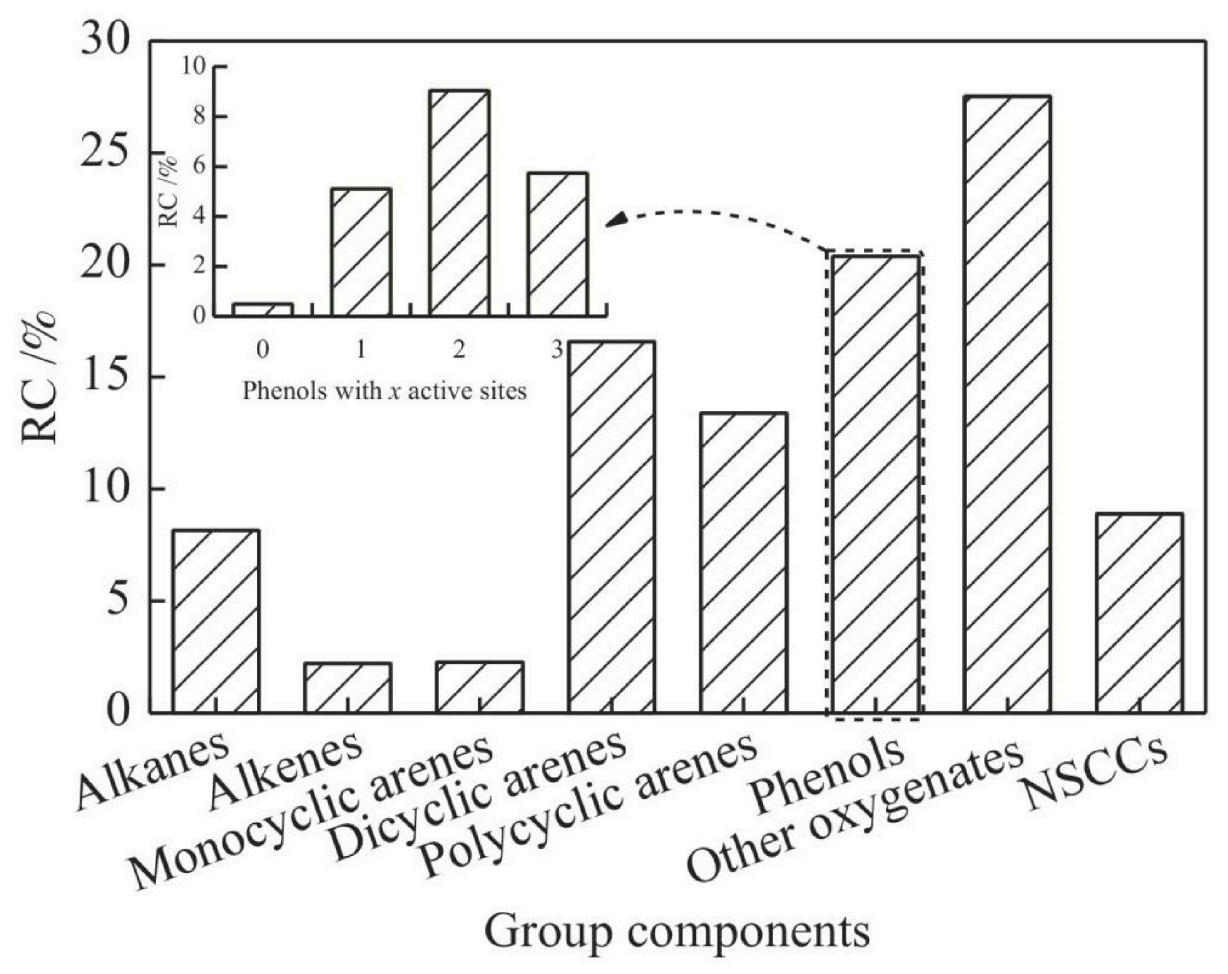
Distributions of group components in low-temperature coal tar [90].

**Figure 13:**
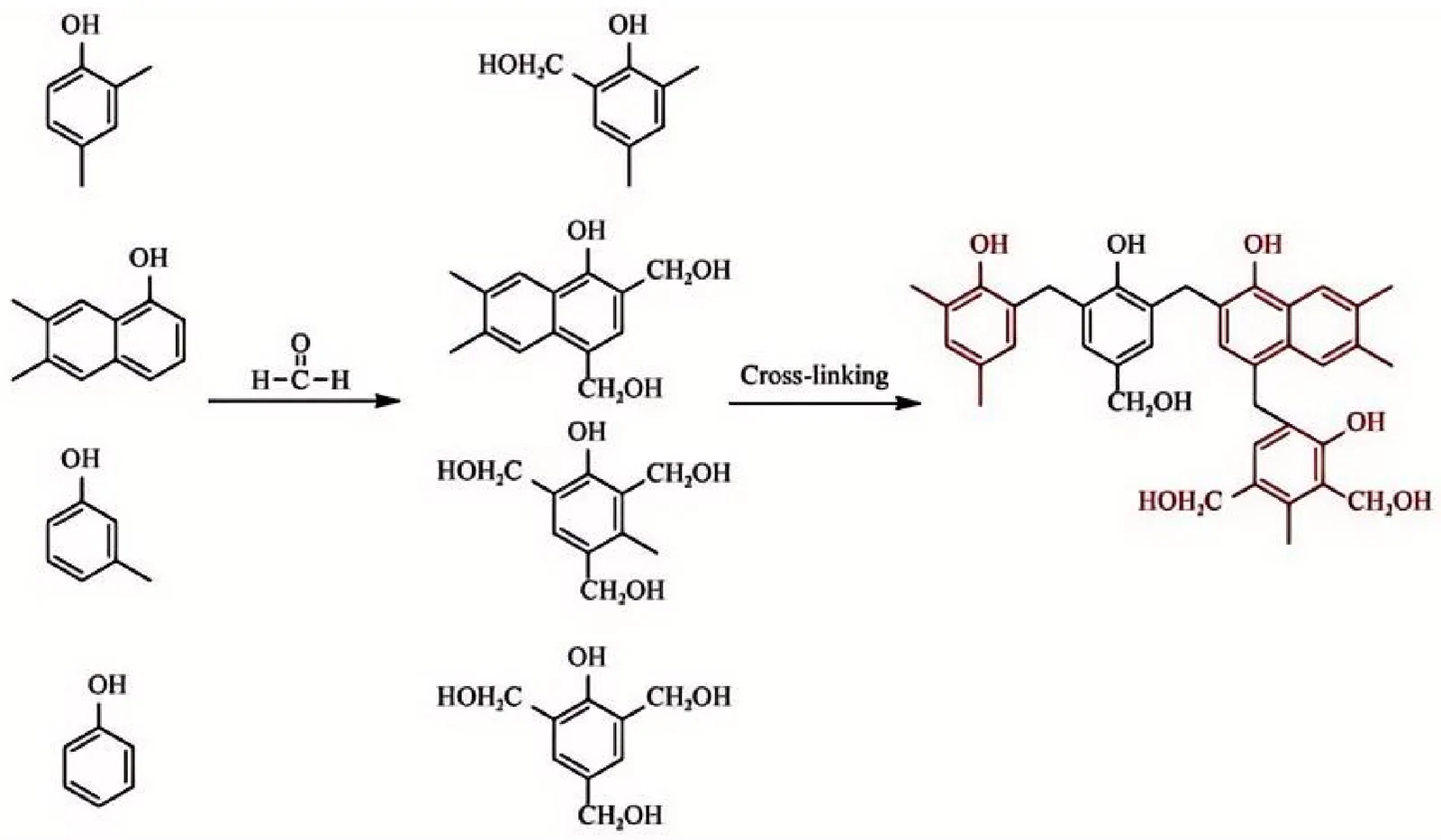
Possible pathways of some components in coal tar participating in polymerization of phenolic resins [90].

(1) Phenolic foam boards are a leading insulation material for core shafts and refuge floors. They offer fire safety, low thermal conductivity, flame retardancy, no dripping, and low toxicity. Compared to polystyrene and polyurethane, they are known as “third-generation polymer foam” with superior fire resistance. Composite-modified phenolic foam achieves an LOI above 35% in vertical burning tests, qualifying as flame-retardant [89]. For cable and pipe shafts, low powdering is critical for long-term reliability. Toughening with isocyanate-functional polyurethane prepolymers reduced the powdering rate from 2.25% to 1.40%. Further modification with closed-cell perlite lowered it to 1.20%. Another study used low-temperature coal tar to replace petroleum-based phenol. The resulting CPF showed powdering rate reductions of 22.9% and 50.8% compared to conventional foam [90] (Figure 12 and Figure 13). Low powdering means less surface flaking and dust generation over time and thermal cycling. This preserves fire barrier integrity and reliability.

(2) In the field of fire protection for steel structures in large public facilities, mature phenolic-based sprayed fireproofing materials have already been adopted internationally. For example, the CAFCO 400 series of cement-based sprayed fireproofing materials produced by Isolatek International is specifically designed to provide structural steel with up to 4 h of fire resistance. With a bond strength exceeding 430 psf (20.6 kPa), it meets the stringent requirements of the International Building Code (IBC) for buildings over 128 m in height. The product’s durability has been verified through 2 bar blast overpressure and subsequent fire exposure testing, making it suitable for areas prone to physical impact, such as mechanical rooms and parking garages. Additionally, its gypsum-based variant, CAFCO 400 AC, also provides up to 4 h of fire resistance rating certification [88,91], making it particularly suitable for fire protection in vertical shaft areas such as elevator shafts.

### 5.2. The Main Challenges Currently Facing Us

(1) Achieving equilibrium between efficiency and expenditure presents a significant challenge. Many highly efficient modification methods such as MXene, functionalized graphene, specialized nanofillers and CVD coatings are associated with high material costs and complicated processes, which makes scaling up for large-scale industrial production difficult.
(2) There is a lack of long-term performance and reliability data. Most studies have focused on short-term combustion tests at the laboratory scale. However, research on long-term service performance remains insufficient. Real-world environments involve multiple coupled factors. These include humid heat, thermal cycling, ultraviolet radiation, and chemical corrosion. Under such conditions, the evolution of flame-retardant properties and resistance to chalking is not well understood. The underlying aging mechanisms also require further investigation. Consequently, there is a lack of reliable models for predicting service life. There is a lack of standardization and consistency in evaluation methods for Disan. There are no internationally recognized, quantifiable standard test methods for evaluating anti-chalking performance, and different research teams employ varying evaluation methods, making it difficult to compare data across studies. This severely hinders technical exchange and product standardization.
(3) With growing pressure for green and sustainable development, the production of traditional phenolic resins, which involves raw materials such as phenol and formaldehyde, raises environmental and health concerns. The sustainable development of the phenolic panel industry now hinges on several key directions. One priority is the development of phenolic resins derived from biomass feedstocks—for instance, substituting phenol with lignin degradation products. Another is the adoption of halogen-free flame retardants with low toxicity. A third is the exploration of effective recycling strategies or controlled degradation methods for panels at the end of their service life. Collectively, these have become essential requirements for the industry’s future.

### 5.3. Outlook on Future Research Directions

The research and development of phenolic boards with high flame retardancy and low charring is a cutting-edge field that involves the interdisciplinary integration of polymer chemistry, composite materials science, combustion science, and solid mechanics. Substantial progress has been made in recent years through advances in several interconnected areas. In resin molecular design, modification with elements such as boron, silicon, phosphorus, and nitrogen has been explored. Flame-retardant systems have evolved to include nanocomposites, intumescent formulations, and bio-based alternatives. Reinforcement interfaces have been optimized via multiscale reinforcement strategies and surface engineering approaches. Forming processes have also been refined through low-pressure impregnation, controlled foaming, and post-treatment protocols. Together, these developments have led to marked improvements in both the quality of the char layer and the material’s ability to maintain structural stability under high-temperature conditions. The increasing sophistication of characterization techniques has also provided powerful tools for a deeper understanding of their performance and mechanisms. Looking ahead, research in this field will continue to advance in multiple directions.

The R&D framework integrates multi-scale simulation (MD, DFT, FEA) with bio-inspired gradient structures to balance strength, toughness, and fire resistance. Smart functionalities (thermal warning, self-healing) are included without sacrificing insulation. Green synthesis uses 30–40% bio-based phenols with reactive diluents to control viscosity and char yield. Standardization addresses dual chalking metrics. Machine learning (graph neural networks) accelerates optimization by integrating composition-process-structure-performance data, including failures. This closed-loop, end-user-driven workflow ensures next-generation fire-resistant panels for aerospace and EV applications.

## Figures and Tables

**Table 1:** Summary of boron and silicon modification effects on phenolic resin char properties.

Modification Type	Refs.	I_D_/I_G_	Char Yield	Hardness/Strength Related Data
Boron-Silicon Cooperative	[26]	2.63 → 1.32 (↓ 49.8%)	77.0%	Tmax ↑ 84 °C
Aryl Boron	[27]	——	70.4%	Flexural Strength ↑, Ablation Rate ↓
Aryl Boron	[28]	——	70.0% (1000 °C, N_2_)	Flexural Strength: 436.8 MPa
Boron Phenolic Curing EP	[29]	——	——	Continuous and Strong Char Layer
Boron Phenolic + PDMS	[30]	——	0.15% → 8.93% (600 °C)	LOI: 32.6%, UL-94 V-0

**↑** = increase **↓** = decrease.

**Table 2:** Comparison of flame retardant systems for phenolic resin composites.

Flame Retardant System	Advantages	Limitations	Best Suited For
Nanocomposite systems	Multi-functional (barrier + reinforcement + smoke suppression); significant char strength improvement	High cost, dispersion challenges, scalability concerns	High-end aerospace and military applications
Intumescent flame retardant systems	Excellent thermal insulation through expansion; self-extinguishing capability	PF compatibility issues, potential migration, reduced mechanical properties	Building insulation, general fire protection
Bio-based flame retardants	Sustainable, low toxicity, renewable feedstock	Lower efficiency than synthetic alternatives, limited long-term data	Green building materials, consumer applications
Hybrid/cooperative systems	Complementary mechanisms, superior overall performance	Complex formulation optimization, potential interactions between components	Applications requiring highest performance levels

## Data Availability

The original contributions presented in this study are included in the article. Further inquiries can be directed to the corresponding author.

## References

[1] Tobiason F.L. (1990). Phenolic resin adhesives. Handbook of Adhesives.

[2] Long S.Y., Qin Y., Liu J.L., Xian X.Q., Zhou L.Q., Lv W.D., Tang P.D., Wang Q.Y., Du Q.S. (2024). Study on the lignin-derived sp2–sp3 hybrid hard carbon materials and the feasibility for industrial production. Sci. Rep..

[3] (2012). Classification for Burning Behavior of Building Materials and Products.

[4] Choi Y.H., Lee S.Y., Kim Y.K., Lee K.M. (2007). Comparative LCA of three types of Interior Panel (IP) in Electric Motor Unit (EMU). J. Korean Soc. Railw..

[5] Folkers J.L. Fiberglass pipe for dry deluge and confined space service. Proceedings of the Offshore Technology Conference.

[6] Akitsu T., Honda A., Imae T., Higashi Y. (2023). Toward flame retardants or thermal stabilizers with new mechanism for polymers. FirePhysChem.

[7] (2018). Fire Classification of Construction Products and Building Elements—Part 1: Classification Using Data from Reaction to Fire Tests.

[8] (2019). Standard Method of Test for the Evaluation of Flammability Characteristics of Exterior Non-Load-Bearing Wall Assemblies Containing Combustible Components.

[9] Kandola B.K., Krishnan L., Ebdon J.R. (2014). Blends of unsaturated polyester and phenolic resins for application as fire-resistant matrices in fibre-reinforced composites: Effects of added flame retardants. Polym. Degrad. Stab..

[10] Xie R.C., Qu B.J. (2001). Synergistic effects of expandable graphite with some halogen-free flame retardants in polyolefin blends. Polym. Degrad. Stab..

[11] Cullis C.F., Hirschler M.M., Tao Q.M. (1991). Studies of the effects of phosphorus-nitrogen-bromine systems on the combustion of some thermoplastic polymers. Eur. Polym. J..

[12] Gallegos I., Varshney V., Kemppainen J., Odegard G.M. (2025). Investigating the structure–property correlations of pyrolyzed phenolic resin as a function of degree of carbonization. Nanoscale Adv..

[13] Zhao Y.H., Li Y., Ma F., Xing X.L., Wang S.J., Hong T., Zhang C.S., Jing X.L. (2024). Optimization of crosslinked network structure of cured phenolic resin with high char yield. Polym. Degrad. Stab..

[14] Nair C.P.R., Bindu R.L., Ninan K.N. (2001). Thermal characteristics of addition-cure phenolic resins. Polym. Degrad. Stab..

[15] Wang S.J., Jing X.L., Wang Y., Si J.J. (2014). High char yield of aryl boron-containing phenolic resins: The effect of phenylboronic acid on the thermal stability and carbonization of phenolic resins. Polym. Degrad. Stab..

[16] Tu J.H., Zhang L.B., Peng J.H., Pu J.Z., Zhang S.M. (2006). Structure evolution of woodceramics by X-ray diffraction and Raman spectroscopy. J. Inorg. Mater..

[17] Ko T.H., Kuo W.S., Chang Y.H. (2001). Microstructural changes of phenolic resin during pyrolysis. J. Appl. Polym. Sci..

[18] Gun’ko V.M. (2021). Polymer adsorbents vs. functionalized oxides and carbons: Particulate morphology and textural and surface characteristics. Polymers.

[19] Tao X., Wang Z.Q., Ma Y.J., Wang M.C. (2026). Mechanical properties and thermal protection performance of fiber reinforced porous phenolic composite prepared by an impregnation and polymerization method. Polym. Compos..

[20] Zhu S.Y., Liu Z.S., Huang B.L., Du K.M., Ma M., Chen S., Shi Y.Q., He H.W., Zhu Y.L., Wang X. (2026). Intrinsic flame retardant polyamide 6 based on high-temperature self-cross-linking carbonization and catalytic synergistic carbonization. ACS Appl. Polym. Mater..

[21] Li S., Han Y., Chen F.H., Luo Z.H., Li H., Zhao T. (2016). The effect of structure on thermal stability and anti-oxidation mechanism of silicone modified phenolic resin. Polym. Degrad. Stab..

[22] Abdalla M.O., Ludwick A., Mitchell T. (2003). Boron-modified phenolic resins for high performance applications. Polymer.

[23] Yuan L., Yang Z.Z., Zeng P., Liang G.Z., Gu A.J. (2024). Behavior and mechanism of flame retardant epoxy resins with intrinsic phosphorus-nitrogen phenolic resin curing agents. Eur. Polym. J..

[24] Wang X.L., Yuan L.L., Zhao H., Ou Y., Gao T., Xu T.T., Chen L.X. (2024). Preparation and properties of phenolic foam modified with boric acid and organosiloxane by supercritical CO_2_ technology. J. Appl. Polym. Sci..

[25] Bao Q.R., Li W.Z., Liu Y., Wang Q. (2019). Preparation and properties of phosphorus- and silicon-modified phenolic resin with high ablation resistance. Polym. Int..

[26] Wang F.Y., Huang Z.X., Qin Y., Li Y.X. (2016). Preparation and thermal stability of heat-resistant phenolic resin system constructed by multiple heat-resistant compositions containing boron and silicon. High Perform. Polym..

[27] Wang S., Jing X., Wang Y., Si J. (2023). The aryl-boron phenolic resins with super ablation properties for resin-transfer molding process of three-dimensional fabric. Polym. Degrad. Stab..

[28] Wang S.J., Jing X.L., Wang Y., Xia Q.C., Si J.J., Zheng W. (2014). Synthesis and characterization of novel phenolic resins containing aryl-boron backbone and their utilization in polymeric composites with improved thermal and mechanical properties. Polym. Adv. Technol..

[29] Deng P., Shi Y., Liu Y.S., Liu Y., Wang Q. (2018). Solidifying process and flame retardancy of epoxy resin cured with boron-containing phenolic resin. Appl. Surf. Sci..

[30] Xie Y.D., Liu C., Wang Y.J., Bao D.X., Yan W., Zhou G.H. (2024). Waterborne polyurethane treated with flame retardant based on polydimethylsiloxanes and boron phenolic resin for improving the char residue and anti-dripping performance. Molecules.

[31] Chen L., Ren D.X., Chen S.J., Pan H., Xu M.Z., Liu X.B. (2019). Copolymerization of phthalonitrile-based resin containing benzoxazine and cyanate ester: Curing behaviors, fiber-reinforced composite laminates and improved properties. eXPRESS Polym. Lett..

[32] Yokoyama N., Park K., Ichikawa T., Takahashi S., Kasemura T. (2009). Morphologies and properties of cured epoxy/NBR containing carboxyl groups (C-NBR)/cycrophenoxyphosphazen (CPP) blends as adhesives for flexible printed circuits (FPCs). J. Adhes. Soc. Jpn..

[33] Li C., Kang N.J., Labrandero S.D., Wan J.T., Gonzalez C., Wang D.Y. (2014). Synergistic effect of carbon nanotube and polyethersulfone on flame retardancy of carbon fiber reinforced epoxy composites. Ind. Eng. Chem. Res..

[34] Zhao J.G., Zeng Q.X., Huang L., Huang L., Xie H. (2026). New resveratrol based Mannich epoxy resin curing agent. Mater. Lett..

[35] Lazko J., Mariage J., Joyet C., Layachi A., Satha H., Dubois P., Laoutid F. (2026). Toward safer and greener insulation: Formaldehyde-free, flame-retardant, and bio-based phenolic foams from tannin and modified-lignin combination. Materials.

[36] Kawalerczyk J., Dukarska D., Barczewski M., Dziurka D., Mirski R. (2023). Optimization of isocyanate content in PF/pMDI adhesive for the production of high-performing particleboards. Polymers.

[37] Wang X.Z., Wang S.Q., Xie X.Q., Zhao L.G., Deng Y.H., Li Y.J. (2017). Multi-scale evaluation of the effects of nanoclay on the mechanical properties of wood/phenol formaldehyde bondlines. Int. J. Adhes. Adhes..

[38] Bakhshi B., Heydarian M. (2021). Study of mechanical, flame, and water stability of phenolic resin/carbon fiber/nanosilica composites. Polym. Compos..

[39] Liu L., Wang Z.Y. (2018). Facile synthesis of a novel magnesium amino-tris-(methylene phosphonate)-reduced graphene oxide hybrid and its high performance in mechanical strength, thermal stability, smoke suppression and flame retardancy in phenolic foam. J. Hazard. Mater..

[40] Guo L.H., Zhang Y.M., Zhang G., Wang Q.H., Wang T.M. (2021). MXene-Al_2_O_3_ synergize to reduce friction and wear on epoxy-steel contacts lubricated with ultra-low sulfur diesel. Tribol. Int..

[41] Meng F.N., Zhang Z.Y., Gao P.L., Kang R.Y., Boyjoo Y., Yu J.H., Liu T.T. (2021). Excellent tribological properties of epoxy—Ti_3_C_2_ with three-dimensional nanosheets composites. Friction.

[42] Nasinathan R., Gnanasekaran A., Rengasamy M., Lakshmanan R., Rajaram K. (2026). Experimental evaluation of a graphite/MXene-based composite phase change material for thermal management in 3s3p lithium-ion battery pack. Energy Technol..

[43] Li H.R., Chen X.P., Sun M.X., Wu H.Z., Tang L.S. (2024). Synergistic fire retardancy of melamine resin modified with pentaerythritol and ammonium polyphosphate in PP. J. Vinyl Addit. Technol..

[44] Wang S., Ma R.M. (2025). Effect of phosphorus-containing silane coupling agents modified ammonium polyphosphate on the flame retardancy and mechanical properties of epoxy resin. J. Appl. Polym. Sci..

[45] Peng H.P., Li D.D., Xu S.W., Zhao Y.G., Lei Y., Zhu H.R., Tan Q.G., Li Z.W. (2025). Application of modified starch @ phenolic resin flame retardant coating on RPUF. J. Appl. Polym. Sci..

[46] Gao Z.W., Lang X.L., Chen S., Zhao C. (2021). Mini-review on the synthesis of lignin-based phenolic resin. Energy Fuels.

[47] Zhang Y., Nan X.T., Zhang S.T., Jia L., Zhu F.B., Yu W.W., Zheng Q. (2025). Enhancing performance of mining phenolic filling materials by tailoring closed cell morphology with fly ash geopolymer. Int. J. Min. Sci. Technol..

[48] Guo Y., Yang Q.S., Huo S.Q., Li J., Jafari P., Fang Z.P., Song P.A., Wang H. (2025). Recyclable fire-retardant bio-based thermosets: From molecular engineering to performances and applications. Prog. Polym. Sci..

[49] Zhou R., Sun X., Xie C.M. (2022). A series of novel flame retardants produced with nanosilica, melamine, and aluminum diethylphosphinate to improve the flame retardancy of phenolic resin. ACS Omega.

[50] Xu J.J., Shao X.D., Lei L.J., Zhang X., Chang J.L., Gao H. (2026). Effect of different reinforcing fibers on the properties of phenolic aerogel composites. Gels.

[51] Xiao W., Wang J. (2025). Silane coupling agent grafted onto the oxidized carbon fiber to improve the interfacial properties of epoxy composites. ECS J. Solid State Sci. Technol..

[52] Wang G.J., Liu Y.W., Guo Y.J., Zhang Z.X., Xu M.X., Yang Z.X. (2007). Surface modification and characterizations of basalt fibers with non-thermal plasma. Surf. Coat. Technol..

[53] Zhang T., Song Y.X., Zhao Y.Q., Zhang B.M. (2018). Effect of hybrid sizing with nano-SiO2 on the interfacial adhesion of carbon fibers/nylon 6 composites. Colloids Surf. A.

[54] Mujika F., Vargas G., Ibarretxe J., De Gracia J., Arrese A. (2012). Influence of the modification with MWCNT on the interlaminar fracture properties of long carbon fiber composites. Compos. B.

[55] Jing X., Ding Y., Chen S., Liu X., Wang Z. (2025). Hybrid effects and failure mechanisms of S-glass/aramid fiber reinforced hybrid composite laminates with wave-transparent. Compos. A.

[56] Zhuang X.W., Li S.H., Ma Y.F., Zhang Z.P. (2011). Preparation and performance research on phenolic insulation foam used low-temperature foaming technology. Adv. Mater. Res..

[57] Bao R., Liu J., Xiao Z., Joshi S.C. (2024). Thermo-chemo-mechanical modeling of residual stress in unidirectional carbon fiber-reinforced polymers during manufacture. Materials.

[58] Ziarati H.B., Fasihi M., Omranpour H. (2020). The effect of resin formulation on the cellular morphology and mechanical properties of phenolic foams. J. Appl. Polym. Sci..

[59] Scheffler T., Englich S., Heyne U., Gehde M. (2016). Effects of post-curing on the thermo-mechanical behavior and the chemical structure of highly filled phenolic molding compounds. Mater. Test..

[60] Fong L., Advani S.G., Peters S.T. (1998). Resin transfer molding. Handbook of Composites.

[61] Sheikh A. (2026). Adaptive control of resin flow during manufacture of composite wind turbine blades. WindEurope Annual Event 2026.

[62] Kirschnick U., Feuchter M., Ravindran B., Salzmann M., Duretek I., Fauster E., Schledjewski R. (2024). Manufacturing bio-based fiber-reinforced polymer composites: Process performance in RTM and VARI processes. Adv. Manuf. Polym. Compos. Sci..

[63] Dereims A., Drapier S., Bruchon J., Léné F., Habraken A.M. (2022). Accurate vacuum assisted resin infusion simulation through a fluid-solid coupled modeling. Mater. Compos..

[64] (2017). Plastics—Determination of Burning Behaviour by Oxygen Index—Part 2: Ambient-Temperature Test.

[65] (2009). Plastics—Determination of Burning Behaviour by Oxygen Index – Part 2: Ambient-Temperature Test.

[66] (2023). Standard for Safety of Flammability of Plastic Materials for Parts in Devices and Appliances.

[67] Kashiwagi T., Cleary T.G. (1993). Effects of sample mounting on flammability properties of intumescent polymers. Fire Saf. J..

[68] Zhang K.T., Wang Z., Nie J.C., Wen F. (2024). Study on combustion characteristics of glass fiber/phenolic resin composites. Heliyon.

[69] Rantuch P., Martinka J., Ház A. (2021). The Evaluation of Torrefied Wood Using a Cone Calorimeter. Polymers.

[70] Moone L., van de Ven I.A.C., Donners M.P.J., Van Durme K., Okhrimenko D.V., van Benthem R.A.T.M., Tuinier R., Esteves A.C.C. (2026). Ageing phenomena in hydrophilic phenolic resin coatings. Polym. Degrad. Stab..

[71] Rinde J.A., Mones E.T., Moore R.L., Newey H.A. (1980). An epoxy resin-elastomer system for filament winding. Polym. Compos..

[72] Shin P.S., Baek Y.M., Kim J.H., Park H.S., Kwon D.J., Lee J.H., Kim M.Y., DeVries K.L., Park J.M. (2018). Interfacial and wetting properties between glass fiber and epoxy resins with different pot lifes. Colloids Surf. A.

[73] Garay A.C., Paese L.T., Souza J.A., Amico S.C. (2015). Studies on thermal and viscoelastic properties of vinyl ester resin and its composites with glass fiber. Rev. Matér..

[74] Ramírez D., Gómez G.F.V., Jaramillo F. (2015). Gel time and polymerization kinetics of unsaturated polyester resin/clay montmorillonite nanocomposites. Polym. Compos..

[75] Zhang Y., Liu Y.D., Jiang D.W., Shalash M., El-Bahy S.M., Wu Z.J., Ren J.N., Guo Z.H., El-Bahy Z.M. (2025). A comprehensive review on modified phenolic resin composites for enhanced performance across various applications. Polym. Compos..

[76] Jafari P., Jafari P., Zhu G.P., Zhu Z.M., Lynch M., Cai T.T., Huo S.Q., Ren J.P., Huang G.B., Song P.A. (2026). AI-aided de novo molecular design of phosphamide for fire-retardant polylactide with enhanced integrated properties. Polym. Degrad. Stab..

[77] Jafari P., Ye G., Wang J., Si J.Y., Qi Y.L., Chen H.Y., Gao C., Song P.A. (2026). Designing flame retardants for epoxy resins with generative AI for preventing battery thermal runaway propagation. J. Mater. Sci. Technol..

[78] Al-Bogami A.S., Maamoun A.A., Kenawy E.-R., Shaker N.O. (2026). Rigid polyurethane foams—based composites for sustainable logistics transportation: Synthesis, structure, multifunctional applications, and artificial intelligence integration. React. Funct. Polym..

[79] Huang S., Luo W., Jiang H., Shi Z.L., Xu R.X., Xie D.L. (2026). Progress and prospects of thermally conductive/flame-retardant integrated polymer composites. Nano Res..

[80] Sabet M., Nawi A.B.A., Prasad D.M.R. (2026). AI-assisted design of halogen-free EVA/Mg(OH)_2_–CNT/GNP nanocomposites for enhanced electrical, thermal, and flame-retardant performance. Polym. Eng. Sci..

[81] Santhosh M.S., Sasikumar R., Khadar S.A., Natrayan L. (2021). Ammonium polyphosphate reinforced E-glass/phenolic hybrid composites for primary E-vehicle battery casings—A study on fire performance. J. New Mater. Electrochem. Syst..

[82] Sakthivel S., Magibalan S., Jeyaprakasam S., Venkatraman J. (2024). Improving fire resistance in E-vehicles: A study on MPP-enhanced S-glass/phenolic hybrid composites. E3S Web Conf..

[83] Swentek I., Ball C.A., Greydanus S., Nara K.R. (2020). Phenolic SMC for Fire Resistant Electric Vehicle Battery Box Applications.

[84] MacDowell H. Design for sustainability in highly flame resistant phenolic sheet molding compound. Proceedings of the CAMX 2023.

[85] (2026). Title 14, Code of Federal Regulations, Part 25, Appendix F—Part I—(a)(1)(i), (a)(1)(ii), (a)(2)(iii), (a)(2)(iv), (a)(3).

[86] Wang L., Hu X., Huang R., Huang M.T., Liu X., Zhou Z.M., Guo P.J., Mao Z.L., Xu X.S., Wang X. (2024). Elastic, antiflaming phenolic aerogels for advanced thermal protection at extreme environments. Compos. Commun..

[87] Sandhya P.K., Sreekala M.S., Thomas S. (2022). Phenolic Based Foams: Preparation, Characterization, and Applications.

[88] (2011). Standard for Safety of Fire Tests of Building Construction and Materials.

[89] Xu W.Z., Zhong D., Chen R., Cheng Z.H., Qiao M.X. (2021). Boron phenolic resin/silica sol coating gives rigid polyurethane foam excellent and long-lasting flame-retardant properties. Polym. Adv. Technol..

[90] Cheng J.Y., Li Z.K., Yan H.L., Lei Z.P., Yan J.C., Ren S.B., Wang Z.C., Kang S.G., Shui H.F. (2023). Preparation and characterization of low-temperature coal tar toughened phenolic foams. J. Fuel Chem. Technol..

[91] (2022). Standard Test Methods for Fire Tests of Building Construction and Materials.

